# Temporal Diffusion Ratio (TDR) for imaging restricted diffusion: Optimisation and pre-clinical demonstration

**DOI:** 10.1016/j.neuroimage.2023.119930

**Published:** 2023-02-05

**Authors:** William Warner, Marco Palombo, Renata Cruz, Ross Callaghan, Noam Shemesh, Derek K. Jones, Flavio Dell’Acqua, Andrada Ianus, Ivana Drobnjak

**Affiliations:** a Centre for Medical Image Computing (CMIC), Computer Science Department, University College London, United Kingdom; b Cardiff University Brain Research Imaging Centre, School of Psychology, Cardiff University, Cardiff, United Kingdom; c School of Computer Science and Informatics, Cardiff University, Cardiff, United Kingdom; d Champalimaud Research, Champalimaud Foundation, Lisbon, Portugal; e AINOSTICS Ltd., Manchester, United Kingdom; f NatBrainLab, Institute of Psychiatry, Psychology and Neuroscience, King’s College London, London, United Kingdom

**Keywords:** Diffusion, Microstructure, Optimization, TDR, Axon diameter, Spinal cord

## Abstract

Temporal Diffusion Ratio (TDR) is a recently proposed dMRI technique (Dell’Acqua et al., proc. ISMRM 2019) which provides contrast between areas with restricted diffusion and areas either without restricted diffusion or with length scales too small for characterisation. Hence, it has a potential for informing on pore sizes, in particular the presence of large axon diameters or other cellular structures. TDR employs the signal from two dMRI acquisitions obtained with the same, large, b-value but with different diffusion gradient waveforms. TDR is advantageous as it employs standard acquisition sequences, does not make any assumptions on the underlying tissue structure and does not require any model fitting, avoiding issues related to model degeneracy. This work for the first time introduces and optimises the TDR method in simulation for a range of different tissues and scanner constraints and validates it in a pre-clinical demonstration. We consider both substrates containing cylinders and spherical structures, representing cell soma in tissue. Our results show that contrasting an acquisition with short gradient duration, short diffusion time and high gradient strength with an acquisition with long gradient duration, long diffusion time and low gradient strength, maximises the TDR contrast for a wide range of pore configurations. Additionally, in the presence of Rician noise, computing TDR from a subset (50% or fewer) of the acquired diffusion gradients rather than the entire shell as proposed originally further improves the contrast. In the last part of the work the results are demonstrated experimentally on rat spinal cord. In line with simulations, the experimental data shows that optimised TDR improves the contrast compared to non-optimised TDR. Furthermore, we find a strong correlation between TDR and histology measurements of axon diameter. In conclusion, we find that TDR has great potential and is a very promising alternative (or potentially complement) to model-based approaches for informing on pore sizes and restricted diffusion in general.

## 1 Introduction

Characterising neural tissue structure at the microscopic scale can provide important information regarding development, plasticity, ageing, as well as inform on the impact of various diseases that affect the central and/or peripheral nervous systems (Lawrence et al., 2021; Tian and Ma, 2017; Dubois et al., 2014; Sexton et al., 2014; Lebel et al., 2010). For example, axon diameter, along with myelin content, is an important property that influences the speed and efficiency of neural communication (Hursh, 1939). Mapping axon diameter can therefore provide insight into basic brain operation, with larger axons being linked to a faster nerve conduction velocity (Hursh, 1939; Ritchie, 1982), as well as insight into the progression of neuronal diseases that alter axon diameter distribution, such as amyotrophic lateral sclerosis (Heads et al., 1991; Cluskey and Ramsden, 2001). On the other hand, cell body (namely soma) sizes are of clinical interest in a range of different conditions: for instance, a decrease in neuronal soma size has been reported in subjects with bipolar disorder (Bezchlibnyk et al., 2007), an increase in motor neuron soma size is present in amyotrophic lateral sclerosis (Dukkipati et al., 2018), while large balloon cells are present in focal cortical dysplasia (Taylor et al., 1971).

Because of these relationships between tissue microstructure and function in the nervous system, techniques for mapping restricted diffusion in the brain and the corresponding characteristic length-scales are of high potential clinical significance. Non-invasive techniques for measuring brain microstructure, such as those developed using diffusion-weighted MRI (dMRI) are especially of interest, as they provide clinically relevant information whilst obviating the need for invasive biopsy and associated risk. dMRI sensitises the signal to the displacement of the water molecules in the tissue and thus provides indirect information about the microscopic tissue organisation (Baliyan et al., 2016; Drake-Pérez et al., 2018; Le Bihan and Iima, 2015) on a scale much smaller than the voxel size. Since dMRI techniques can report on representations of diffusion, to enhance specificity, modelling strategies can be employed - under strict assumptions - to inform in a quantitative way on the underlying microstructure. Towards this goal, several methods have been developed in the past to characterise restricted diffusion in tissue and to map cellular sizes, both in white matter (WM) and grey matter (GM).

In white matter, mapping axon diameter has been the focus of several dMRI studies over the last decades (Stanisz et al., 1997; Assaf et al., 2008; Alexander et al., 2010; Harkins et al., 2021; Veraart et al., 2020; Kakkar et al., 2018; Huang et al., 2020; Duval et al., 2015; Grussu et al., 2019; Sepehrband et al., 2016; Fan et al., 2020; Barazany et al., 2009; Zhang et al., 2011; Ong and Wehrli, 2010; Shemesh, 2018; Xu et al., 2014; Bar-Shir and Cohen, 2008; Shemesh et al., 2015). Some approaches employ biophysical models of the tissue, for example, representing intra-axonal signal as diffusion restricted in infinitely-long non-permeable cylinders, and extra-axonal signal as Gaussian hindered diffusion (Assaf et al., 2008; Alexander et al., 2010). Then, the tissue model is fitted to a rich dMRI acquisition, usually with several diffusion-weighting amplitudes (‘shells’) and diffusion times for each shell, to estimate summary statistics drawn from the apparent axon diameter distributions. Another recently proposed approach is to use diffusion measurements with ultra-high diffusion weighting (e.g. *b* > 6 ms/*μ*m^2^) to map axon diameter sizes based on the departure from the power law expected for diffusion inside cylinders with negligible diameters (i.e. ‘sticks’), however this approach requires at least one b-value much greater than 10 ms/ *μ*m^2^, leading to lower resolution data on account of longer TEs and the lower SNR at these high b-values (Veraart et al., 2020). To improve sensitivity to axon diameter (Drobnjak et al., 2016), other approaches go beyond the conventional single diffusion encoding acquisitions and also use oscillating gradient waveforms that can access much shorter diffusion times, either included in a modelling frame-work (Kakkar et al., 2018; Siow et al.) or used to contrast measurements with different diffusion times and frequencies (Harkins et al., 2021; Does et al., 2003; Wu et al., 2019). It has been shown in multiple works (Drobnjak et al., 2016; Nilsson et al., 2017 ; Paquette et al., 2021) that measurements of axon diameters and/or diameter distributions are extremely challenging, regardless of the dMRI sequence used, especially when considering standard gradient amplitudes available on clinical scanners. The biggest challenge is the low inherent sensitivity of the signal towards axons of small diameter, with the vast majority of contrast being produced by large axons from the tails of most biological axonal distributions (Drobnjak et al., 2016; Nilsson et al., 2017).

In grey matter, mapping cell soma sizes is a more recent endeavour in dMRI, with several studies showing the benefits of including cell soma in microstructure models (Palombo et al., 2020; Ianus et al., 2021; Schiavi et al., 2022; Afzali et al., 2020), suggesting mapping restricted spaces such as cell soma is a good additional target for new microstructure imaging techniques. However, it has also been recently shown that, compared to WM, GM may be characterised by faster multi-compartmental exchange of water molecules (Jelescu et al., 2022; Olesen et al., 2022; Olesen et al., 2022), which represents an extra challenge for the biophysical modelling of dMRI measurements and the unbiased estimation of restricted diffusion in GM.

Furthermore, studying the time dependence of the diffusion signal, as well as of estimated parameters such as diffusion and/or kurtosis tensors, can provide additional information about the tissue characteristics and inform about the type of diffusion processes. For instance, measuring the diffusion time dependence of the diffusion and kurtosis tensors can help to differentiate between the effects of restricted diffusion and inter-compartmental exchange (Jelescu et al., 2022; Olesen et al., 2022; Olesen et al., 2022; Reynaud, 2017; Lampinen et al., 2021; Assaf et al., 2000; Jespersen et al., 2018; Lee et al., 2020; Aggarwal et al., 2020; Xu et al., 2016), with various applications for brain and body imaging. Although of great interest, characterising both the time dependence and b-value dependence (e.g. to estimate diffusion and kurtosis tensors) requires many measurements and cannot currently be performed in a practical amount of time for clinical applications.

A common feature of the vast majority of dMRI techniques developed for characterising both WM and GM microstructure is the use of biophysical models to describe the relationship between the measured dMRI signals and the underpinning tissue microstructure. Imaging markers of histologically relevant features, such as axon diameter, are then inferred by fitting such biophysical models. Although very successful for many applications (Alexander et al., 2019; Novikov et al., 2018), this paradigm has some major limitations: e.g. it relies on strong assumptions on the relevant features characterising the underlying tissue structure, the models used are often overly simplistic, and it is prone to ambiguities of results interpretation due to inherent model degeneracies. Other research also exists, which aims to avoid modelling approaches and use diffusion time dependence to characterise tissues while keeping other diffusion parameters the same, such as the studies on diffusion dispersion rate (Xu et al., 2016), ΔfADC (Wu et al., 2014), ΔD⊥ (Harkins et al., 2021), and SSIFT (Devan et al., 2022). However, these studies do not use large b-values and hence the signal is sensitive to both restricted and non-restricted tissues making it hard to apply for mapping axon or soma diameters.

To bypass some of these limitations, Dell’Acqua et al. (2019) recently in a preliminary study presented at ISMRM 2019, introduced the Temporal Diffusion Ratio (TDR), a model-free dMRI approach to characterise restricted diffusion inside large axons using two b-value shells with different gradient timings. Specifically, TDR employs dMRI measurements with a very high b-value (above 7 ms/ *μ*m^2^) to suppress fast and assumingly extra-axonal diffusion, and contrasts the signal from two shells at the same b-value obtained with different diffusion times and gradient settings. The advantages of TDR are that it employs standard PGSE diffusion sequences widely accessible on commercially-available MRI systems without any specialist programming, that it does not make any assumptions on the underlying tissue structure, and that it does not require any model fitting, avoiding issues related to model degeneracy (Paquette et al., 2021; Jelescu et al., 2016).

In this paper we introduce and validate for the first time the unique potential of the TDR contrast to inform on restriction in tissue, building on the preliminary work by Dell’Acqua et al. (2019). We optimise the TDR methodology and demonstrate it in a pre-clinical study. We extend the TDR approach from characterising cylindrical restrictions (e.g. axons, as it was originally introduced in Dell’Acqua et al. (2019)), to include both cylindrical and spherical restrictions (e.g., neurites and cellular bodies), allowing us to understand the TDR values observed in a wide range of tissue types. We employ simulations to optimise the gradient shapes to increase the TDR sensitivity to a wide range of axon diameter distributions, modelled as cylinders, as well as cell body sizes, modelled as spheres. We also investigate the effect of the Rician noise floor on the TDR values and present a strategy for maximising TDR contrast by using a subset of the full gradient directions. Finally, we demonstrate the optimised TDR strategies as well as the relationship between TDR and axon diameter using ex-vivo rat spinal cord data.

## 2 Methods

### 2.1 TDR approach

TDR is computed by contrasting two spherically-averaged dMRI signals with the same b-value, each collected with a different set of diffusion gradient parameters (e.g. diffusion time, gradient strength), following the expression:

$$TDR=(S_{2}-S_{1})/S_{2},$$

where the sequence parameters used to generate S_1_ and S_2_ are chosen so that S_2_ > S_1_ for restricted diffusion. Note that, given that the sequences have the same b-value, Gaussian diffusion would result in equal signal values and no TDR contrast, while in restricted diffusion (and any time dependent process) the specific values of S_1_ and S_2_, as well as TDR contrast, depend on the pore size and diffusion time. In the original implementation of TDR, the two shells are acquired using Single Diffusion Encoding (SDE) sequences, with fixed and equal gradient pulse duration *δ*, and different diffusion times Δ, one short and one long (Dell’Acqua et al., 2019). Then, the normalised spherical mean diffusion signals, S^mean^, are calculated either averaging over gradient directions uniformly sampled over a sphere (e.g. using a HARDI acquisition), or using spherical harmonics keeping order zero. TDR contrast is then calculated based on the spherical mean data, to diminish the effect of fibre orientation distribution: (Callaghan et al., 1979)

$$TDR=\frac{S_{2}^{mean}-S_{1}^{mean}}{S_{2}^{mean}}.$$

Here, we calculate the spherical mean signals S_1_^mean^ and S_2_^mean^ by averaging across gradient directions, and hence get TDR as following:

$$TDR=\frac{\sum_{i=1}^{N}S_{2,i}-\sum_{i=1}^{N}S_{1,i}}{\sum_{i=1}^{N}S_{2,i}},$$

where *N* is the total number of uniformly distributed gradient directions in the HARDI acquisition, *S*_1,*i*_ is the signal acquired with the *i* th gradient direction and gradient waveform used to create *S*_1_, while *S*_2,*i*_ is the signal acquired with the *i*th gradient direction and gradient waveform used to create *S*_2_.

To illustrate the rationale behind the TDR contrast, originally proposed for characterising white matter tissue, we consider a simple tissue model consisting of two compartments: intra-axonal signal is modelled as restricted diffusion inside cylinders and contributes to TDR contrast, and extra-axonal signal is modelled as Gaussian diffusion (Veraart et al., 2019; Skinner et al., 2015) and does not contribute to the TDR contrast.

**Fig. 1:**
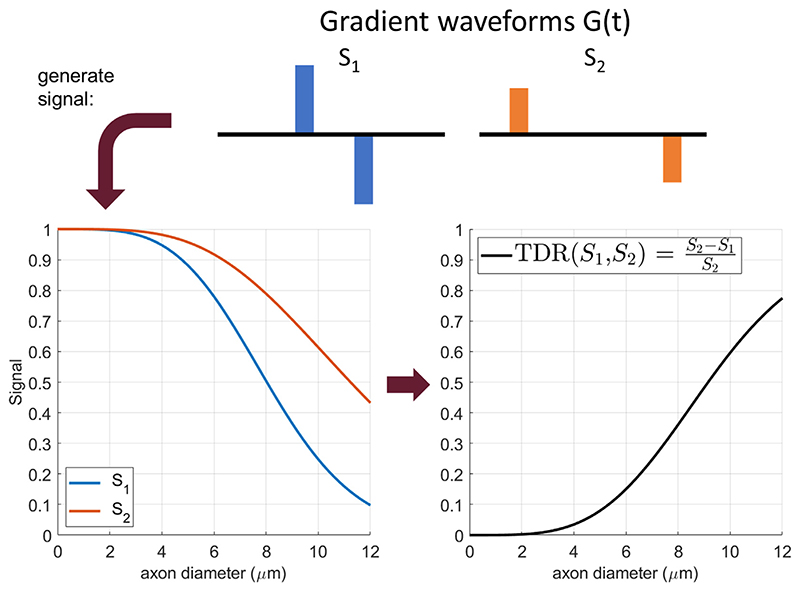
An illustration of the TDR contrast, as proposed in the original study (Dell’Acqua et al., 2019). Both signals (left) are simulated at a b value of 8 ms/*μ*m^2^, with Δ = 21 ms, *δ* = 9 ms, *G* = 276.8 mT/m for S_1_ and Δ = 55 ms, *δ* = 9 ms, *G* = 162.9 mT/m for S_2_, respectively. The relative difference between the two signals produces the TDR contrast on the right, that is monotonically increasing with pore size for the range considered here.

Fig. 1 shows the signal attenuation and TDR values for spins restricted inside a cylinder as a function of diameter when the diffusion gradients are perpendicular to the fibres. The signals S_1_ and S_2_ correspond to sequences with the same b-value (large enough to attenuate extra-cylindrical diffusion) but different gradient waveforms, each yielding a different attenuation for restricted diffusion. For the range of sizes considered here, the difference between S_2_ and S_1_, and thus the TDR contrast (right) increases with the cylinder diameter, here proxy for the axon diameter.

In reality, each white matter voxel contains a range of axon diameters that contribute to the signals S_1_ and S_2_. In this case the TDR contrast is effectively the integral over the distribution of axon diameters. Hence, given that the difference between S_1_ and S_2_ increases with axon diameter, TDR contrast will be higher in voxels containing many large axons. In other words, the TDR contrast is mostly sensitive to large axons and therefore has the potential to map regions where there are larger axons.

In this study, we propose to optimise the dMRI gradient waveform to maximise TDR contrast. The principle driving our optimization is that the best contrast (i.e. maximum TDR value) is found for the smallest S_1_ and largest S_2_ values, for a given b value and restriction size. Hence, we perform numerical simulations to find the actual optimal gradient waveform that maximises TDR contrast given specific hardware constraints for different substrates. We also analyse the TDR contrast in spherical pores, that have been included as a signal compartment when modelling grey matter (Palombo et al., 2020) as well as other tissue types, for instance tumours (Panagiotaki et al., 2014; Roberts et al., 2020).

### 2.2 Simulations

In this study we perform simulations to design optimised SDE acquisitions that maximise TDR contrast. In the first set of simulations we optimise the gradient waveform, then we explore the intensity and the potential of TDR as an imaging contrast.

Simulations are generated using the Microstructure Imaging Sequence Simulation Toolbox (MISST, version 0.93) (Ianuş et al., 2016), a diffusion MRI simulator that calculates the signal attenuation for various restricted geometries and different gradient waveforms using the matrix formalism (Callaghan, 1997; Drobnjak et al., 2010; Drobnjak et al., 2011). Code for MISST is available at http://mig.cs.ucl.ac.uk/index.php? *n*=Tutorial.MISST. Code written by the authors for the optimisation and analysis is also available and can be found at the link in the footnote.^3^

#### 2.2.1 Simulation 1: optimising gradient waveforms for TDR

**Fig. 2:**
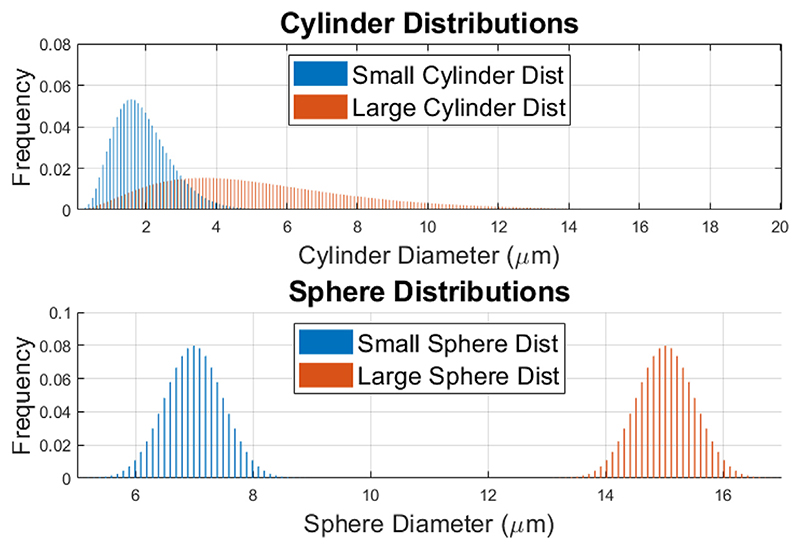
An illustration of the substrate distributions used: two gamma distributions of parallel cylinders (small: mean = 1.93 *μ*m, std = 0.81 *μ*m; large: mean = 5.33 *μ*m, std = 3.00 *μ*m) and two normal distributions of spheres (small: mean = 7 *μ*m, std = 0.5 *μ*m; large: mean = 15 *μ*m, std = 0.5 *μ*m).

In our first simulation, we optimise the gradient waveform for each of the two SDE sequences to maximise TDR contrast. We consider substrate configurations for two different geometries: (a) distributions of parallel impermeable cylinders (mimicking axons), and (b) distributions of impermeable spheres (mimicking cell bodies), illustrated in Fig. 2. For each of these two geometries, we consider two diameter distributions, one large and one small. For cylinders we use large and small gamma distributions to mimic axons typically found in the spinal cord (Grussu et al., 2019; Duval et al., 2019) (mean = 5.33 *μ*m, std = 3.00 *μ*m) and corpus callosum (Grussu et al., 2019; Aboitiz et al., 1992) (mean = 1.93 *μ*m, std = 0.81 *μ*m), respectively. For spheres we use large (Rajkowska et al., 1998; Palombo et al., 2021) (mean = 15 *μ*m, std = 0.5 *μ*m) and small (Palombo et al., 2021; Lackey et al., 2018; Houston et al., 2017) (mean = 7 *μ*m, std = 0.5 *μ*m) normal distributions to mimic cell somas typically found in mammalian brains. The intrinsic diffusivity for all pores is set to 2 *μ*m^2^ /ms, a value typically used for tissue at 37 °C and close to the parallel diffusivity associated with the intra-axonal compartment in ex-vivo spinal cord (Olesen et al., 2021). The signal from extra-axonal space, as well as any exchange effects are assumed to be negligible for the TDR contrast.

We assume the same b-value for both SDE sequences in order to ensure the diffusion contrast from Gaussian diffusion is the same. We set b to a very high value, specifically *b* = 8 ms/ *μ*m^2^ as in the initial work introducing TDR (Dell’Acqua et al., 2019), in order to eliminate the signal that comes from the extra-axonal space and free water. For comparison, in the Supplementary Material, we additionally include results for optimisations performed at *b* = 20 ms/ *μ*m^2^ (Veraart et al., 2020), which ensures an even higher signal attenuation in the extra-axonal space. TDR is calculated from normalised diffusion signals averaged over 60 gradient directions uniformly sampled over a sphere as in the original implementation of TDR (Dell’Acqua et al., 2019).

Optimisation is done using a range of simulations performed with MISST and spanning a large space of sequence parameters, for four high performance animal and human scanner hardware constraints: (1) *G* < 600 mT/m and Δ+*δ* < 45 ms corresponding to a typical pre-clinical scanner; (2) *G* < 2700 mT/m, Δ+*δ* < 45 ms corresponding to a high-gradient pre-clinical system; (3) *G* < 300 mT/m, Δ+*δ* < 80 ms corresponding to the (human MRI) Connectom scanner (Siemens Healthineers); 4) *G* < 80 mT/m, Δ+*δ* < 80 ms corresponding to a high performance clinical scanner. Optimisation is automated using the interior point algorithm as implemented in the MATLAB fmincon function, with Δ, *δ* and G values for each of the two sequences as linked variables under investigation. The interdependence of these variables means we are ultimately optimising over a 4-dimensional space [Δ_1_, *δ*_1_, Δ_2_, *δ*_2_] with Δ and *δ* for each of the two sequences determining the appropriate G-values. Note that to keep consistent with previous TDR work and widely available pre-clinical and clinical settings, we only considered SDE waveforms (i.e. other waveforms such as double diffusion encoding, oscillating gradients or b-tensor encoding were not considered here). Moreover, the gradient slew rates are considered infinite, a good approximation for the preclinical scanner used in our experiments that has a fixed rise time of 0.1 ms (leading to slew rates up to 25,000 mT/ms). Other gradient settings for clinical scanners, as well as the effect of a finite slew rate (set to 200 mT/m to account for physiological constraints) are explored in Supplementary Material Sections S1 and S2.

We then evaluate the performance of the optimised gradient wave-forms against a set of gradients chosen according to the previously published strategy for TDR (Dell’Acqua et al., 2019), namely both sequences have the same, short gradient duration, and only the diffusion time is varied: S_2_ has a long diffusion time and S_1_ has a short diffusion time. The exact values of *δ*, Δ and G were chosen according to the scanner constraints (1–3), with the minimum possible *δ* and Δ for S_1_ and maximum possible Δ for S_2_. As these sequences were not determined based on full scale optimisation, we will refer to them as “non-optimised” wave- forms/sequences.

#### 2.2.2 Simulation 2: effect of restriction size on TDR

In the second simulation we use optimised sequences obtained from simulation 1 to evaluate the TDR contrast over a wide range of pore size distributions.

We consider both distributions of cylinders and spheres to model restrictions in both white matter and grey matter, with size distributions commonly found in tissue (Grussu et al., 2019; Palombo et al., 2020; Aboitiz et al., 1992; Rajkowska et al., 1998; Palombo et al., 2021; Lackey et al., 2018; Houston et al., 2017). Cylinder sizes follow a gamma distribution (Alexander et al., 2010; Grussu et al., 2019), whilst sphere sizes follow a normal distribution (Rajkowska et al., 1998). For cylinders, the gamma distributions are truncated at 20 *μ*m to match realistic values from the tissue; thus, combinations of parameters where this truncation changes the mean by more than 10% are not considered. For all substrates, intrinsic diffusivity is set to 2 *μ*m^2^ /ms and extra-cellular space is neglected. These simulations are run without noise to illustrate the maximum potential of TDR as an imaging contrast.

#### 2.2.3 Simulation 3: effect of gradient directions and noise on TDR

In the third simulation, we investigate the effect of the Rician noise floor on TDR values. To this end, we consider different fibre configurations: one, two and three fibre bundles consisting of parallel cylinders with the same diameter distributions (Gamma distribution with mean = 5.33 *μ*m, std = 3.00 *μ*m) with separate fibre bundles crossing at right angles (in the case of two and three fibres), as well as spherical pores (Gaussian distribution with mean = 7 *μ*m, std = 0.5 *μ*m), and we simulate the signals using the optimised sequences from simulation 1. Then, for each measurement, we consider Rician noise at SNR levels of 20 and 50 in the non-diffusion weighted images, typical for clinical and pre-clinical acquisitions (Tian et al., 2022; Ianuş et al., 2022), as well as noise free signals, i.e. SNR ∞. Finally, we explore different numbers of gradient directions (30 and 60).

We also look at the effect of the orientation distribution of gradient directions included when calculating TDR. In the previous work (Dell’Acqua et al., 2019) S_1_ and S_2_ are calculated as the signal average over a set of uniformly distributed gradient directions. However, in white matter, due to the fast signal decay in certain directions (e.g. parallel to the fibres), the contribution to the direction-averaged signal of these measurements will carry little to no extra information about the axon diameter and could potentially introduce bias to the TDR estimation, due to the noise floor. Here we investigate this effect and its impact on TDR.

### 2.3 Preclinical experiments

All animal studies were approved by the competent institutional and national authorities, and performed according to European Directive 2010/63.

The preclinical experiments aim to investigate TDR contrasts in exvivo rat spinal cord, to validate our optimisation, both in terms of gradient wave-forms as well as the number of HARDI directions employed, and the relationship between TDR and axon diameter in different WM ROIs.

The data and code from the preclinical experiments is available at https://github.com/andrada-ianus/TDR_study.git.

#### 2.3.1 Data acquisition

One rat spinal cord was extracted via transcardial perfusion with 4% Paraformaldehyde (PFA). After extraction, the spinal cord was immersed in a 4% PFA solution for 24 h, and then washed in a Phosphate-Buffered Saline (PBS) solution for 24 h. Two sections of cervical spinal cord were cut and placed separately in 5 mm NMR tubes filled with Fluorinert (Sigma Aldrich, Lisbon, PT). The samples were imaged on a 16.4 T Bruker Aeon Ascend scanner (Bruker, Karlsruhe, Germany) equipped with a 5 mm birdcage coil and gradients capable of producing up to 3 T/m in all directions.

#### 2.3.2 Imaging protocol

Diffusion MRI datasets for TDR were acquired using a SDE-EPI sequence with the following parameters: TE = 50 ms, TR = 2 s, 16 averages, slice thickness = 0.5 mm, 5 slices, in plane resolution = 0.09 × 0.09 mm^2^, matrix = 60 × 46, FOV = 5.4 × 4.15 mm^2^, Partial Fourier = 1.12. The EPI acquisition bandwidth was 400 kHz and data was acquired in a single shot using double sampling, with a total acquisition time of 1 h 50 m for each combination of G_max_ and b-value.

In terms of diffusion weighting, the TDR acquisition was performed for two fixed b-values of 8 and 20 ms/ *μ*m^2^. The b-values were chosen to be similar to those proposed by Dell’Acqua (Dell’Acqua et al., 2019) for TDR and by Veraart et al. (2020) for axon diameter imaging, respectively. For each b-value we consider two scenarios: (1) the sequence parameters were chosen so that the maximum gradient strength was limited to 600 mT/m, a value available on many preclinical systems, and (2) the maximum gradient strength was 2500 mT/m, close to the maximum available on this gradient system. For each scenario, we considered wave-forms with the same gradient duration and different diffusion times, as originally proposed in (Dell’Acqua et al., 2019), referred to as the non-optimised protocol, as well as the optimised wave-forms proposed in this study. Each shell was acquired with 10 b0 values and 60 diffusion directions each, and the specific timing parameters for the non-optimised and optimised protocols are provided in Section 3.2.

The data was acquired with an in-house implementation of the sequence which loops through the different diffusion times in order to avoid any potential signal differences caused by sequence adjustments. The sequences implemented in PV6.0.1 are available upon request.

#### 2.3.3 Data analysis

Pre-processing: Complex data were denoised using the MP-PCA approach (Veraart et al., 2016) following the steps described in (Ianuş et al., 2022) to account for the effect of Partial Fourier acquisition in the data, and the magnitude was computed. Then the data was normalised for each shell.

#### 2.3.4 ROI analysis

For selected WM tracts with different axonal properties (Vestibulospinal (VST), Reticulospinal (ReST), Rubrospinal (RST), dorsal corticospinal (dCST), Funiculus Cuneatus (FC) and Funiculus Gracilis (FG)), the averaged TDR values were compared with axon diameters estimated from quantitative histology reported in the literature (Dula et al., 2010).

### 2.4 Effect of extra-axonal space and orientation dispersion

To further explore different confounding factors that might affect TDR, we employ simulations to study the effect of extra-axonal space and fibre dispersion.

#### 2.4.1 Extra-axonal space

Monte Carlo simulations were performed in Camino (Hall and Alexander, 2009) for the same sequences employed in the preclinical experiments and six substrates consisting of randomly packed cylinders with intra-axonal fraction of 0.6 and Gamma distributed diameters with the same mean and standard deviation as reported for the spinal cord ROIs: 4.47 ± 0.51 *μ*m (VST), 2.22 ± 0.21 *μ*m (ReST), 3.39 ± 0.47 *μ*m (RST), 3.73 ± 0.36 *μ*m (FC), 1.16 ± 0.1 *μ*m (dCST), 1.80 ± 0.13 *μ*m (FG). Simulations were run with spins distributed either uniformly (200,000 spins) or only in the intra-axonal space (120,000 spins). We fix the diffusivity to 2 *μ*m^2^/ms according to the study from Olesen et al. (2021) who estimated a diffusivity value ~2 *μ*m^2^/ms associated with the intra-axonal compartment in the ex-vivo spinal (following the same fixing procedure as ours). We also perform simulations in more realistic substrates using the ConFIG frame-work (Callaghan et al., 2020; Callaghan et al., 2021) and report these results in the Supplementary Material Section S5.

#### 2.4.2 Fibre dispersion

Numerical simulations using MISST were run for a model of dispersed cylinders with a Gamma distribution of diameters and a Watson distribution of orientations (Watson, 1965; Zhang et al., 2012). The simulations were performed for the same sequences and diameter distributions as above, and three different concentration parameters of the Watson distribution: *k* = 1 yields highly dispersed orientations, *k* = 6 yields an orientation dispersion that has been reported in WM, and *k* = 100 yields close to parallel cylinders.

## 3 Results

### 3.1 Simulations

#### 3.1.1 Simulation 1: optimising gradient waveforms for TDR

This section presents the optimisation results of the TDR contrast. As described in Methods, both the first and the second shell of the “optimised protocol” were optimised to maximise TDR contrast across the whole space of possible waveform parameters such as gradient duration, diffusion time and gradient strength. The boundaries of the search space are fixed to pre-clinically achievable values and b-value is fixed. The two shells of the “non-optimised protocol” were adopted from the previously published preliminary work (Dell’Acqua et al., ISMRM 2019) and those were selected empirically and from theoretical intuition, but were not optimised. Here we evaluate which protocol provides stronger TDR contrast.

For the optimised protocol, the space of parameters we explore is typical for pre-clinical scanners, namely *G* < 600 mT/m and Δ+*δ* < 45 ms - presented here. We also do optimisation for other scanner settings: *G* < 2700 mT/m, Δ+*δ* < 45 ms (corresponding to a high-gradient pre-clinical system), *G* < 300 mT/m, Δ+*δ* < 80 ms, (corresponding to the Connectome scanner), and *G* < 80 mT/m, Δ+ *δ* < 80 ms (corresponding to a high performance clinical scanner) - presented in Supplementary Material, Section S1. We provide optimisation results for two different b-values: *b* = 8 ms/ *μ*m^2^ (results presented in this section and Supplementary Material Section S1) and *b* = 20 ms/ *μ*m^2^ (results presented and discussed in Supplementary Material, Section S3).

**Fig. 3:**
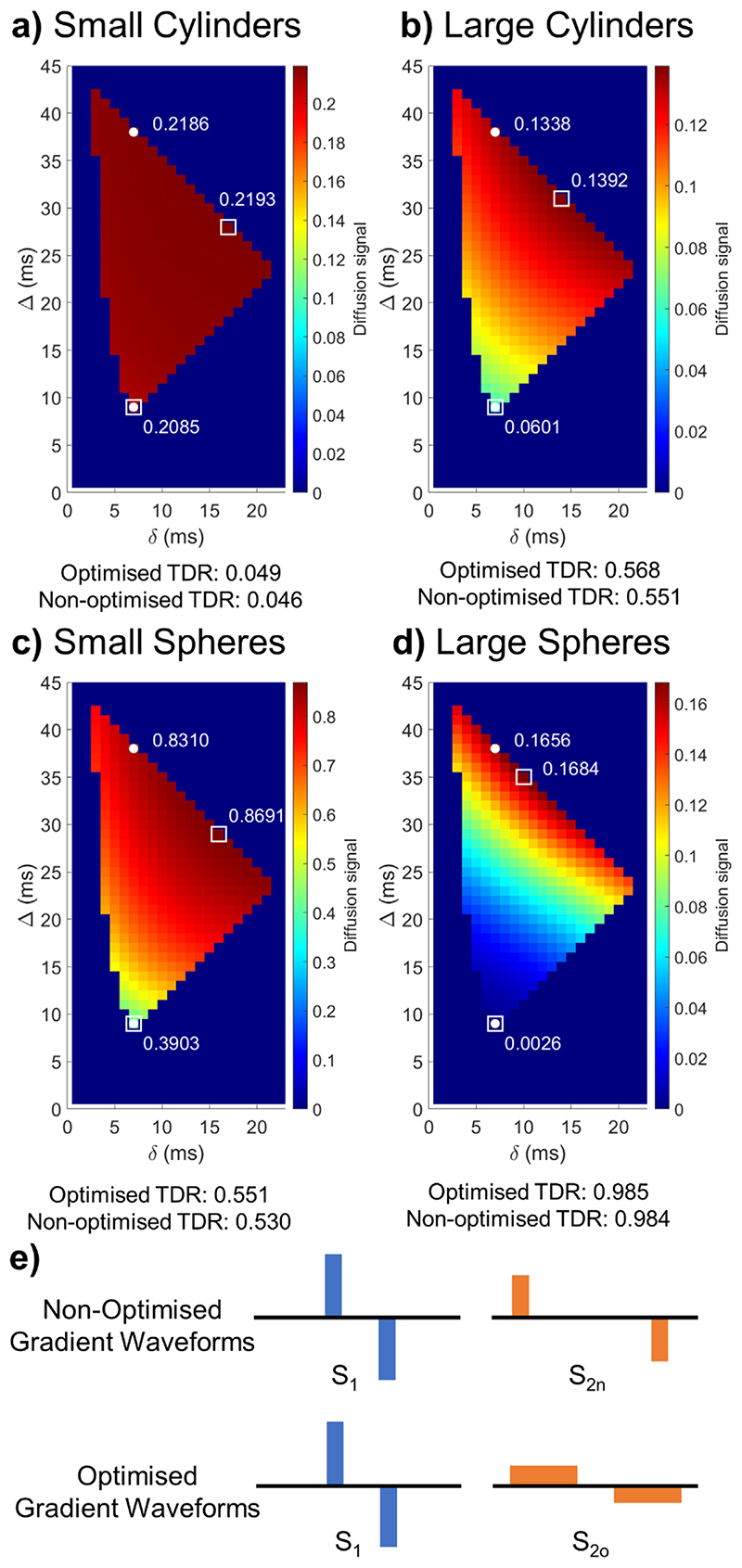
Optimisation results maximising TDR for *G* < 600 mT/m, Δ+*δ* < 45 ms and *b* = 8 ms/*μ*m^2^. (a-d) Maps showing the diffusion weighted signal averaged over the 60 uniformly distributed directions for sequences with various *δ*/Δ combinations for the substrates illustrated in Fig. 2. White markers indicate the non-optimised (circle) and optimised (square) sequences, respectively. The optimised sequences provide larger signal differences between S_1_ and S_2o_ compared to between S_1_ and S_2n_, and higher TDR values. In each plot, colours are scaled so that a diffusion signal of 0 is blue, and the maximum diffusion signal obtained is red; the colour range displayed in each plot gives an idea of the maximum TDR possible. (e) Schematic representation of nonoptimized and optimised gradient shapes. These figures show that optimised TDR maximises the difference between the signal from the two acquisitions, using sequences with different pulse shapes. Equivalent figure for *b* = 20 ms/ *μ*m^2^ is Fig. S2 presented in Supplementary Material.

Fig. 3a illustrates diffusion weighted signal values averaged over 60 uniformly distributed gradient directions for *G* < 600 mT/m, Δ+*δ* < 45 ms: and a range of different combinations of gradient durations and diffusion times and a substrate consisting of small cylinders. Signal values ranging from low (blue) to high (red) values are shown, and TDR is calculated for each pair. In order to find the combination of diffusion sequence parameters that maximises TDR, the optimisation looks for values for S_1_ and S_2_ that are most different from one another. The corresponding, optimal, pair of gradient waveform parameters (squares) has one gradient waveform with short duration and diffusion time and the other with long duration and diffusion time. The short duration waveform is the same as the short diffusion time sequence in the “non-optimised” approach (circles), while the second optimal waveform has much longer gradient duration than any sequence in the “non-optimised” approach, where the diffusion duration is kept constant between the two waveforms and only diffusion time has changed (Fig. 3e).

Similar results are obtained for other substrates as well: large cylinders Fig. 3b, small spheres Fig. 3c and large spheres Fig. 3d - although for spheres with large diameters the optimal duration of the long gradient sequence is slightly shorter. The exact values for all optimised waveform parameters as determined through non-linear optimisation are:

- small cylinders: S_1_ : Δ = 8.9 ms, *δ* = 6.9 ms; S_2o_: Δ = 28.5 ms, *δ* = 16.5 ms
- large cylinders: S_1_: Δ = 8.9 ms, *δ* = 6.9 ms; S_2o_: Δ = 31 ms, *δ* = 14.1 ms
- small spheres: S_1_: Δ= 8.9 ms, *δ* = 6.9 ms; S_2o_: Δ= 29 ms, *δ* = 15 ms
- large spheres: S_1_ : Δ= 8.9 ms, *δ* = 6.9 ms; S_2o_: Δ= 35 ms, *δ* = 9.9 ms

In addition to the specific substrates shown in Fig. 3, we have also performed the optimisation for other substrates, consisting of both cylinders and spheres. Whilst distributions that include very large pores can have different optimal sequence shapes, in all cases considered, increasing *δ* for the S_2o_ shell improves the contrast compared to the non-optimised version of TDR (data not shown). When we repeated the simulations above for *b* = 20 ms/ *μ*m^2^, we found extremely similar optimised sequence patterns and TDR values for the different substrates as presented in Fig. S3 in Supplementary Material.

Furthermore, we have done optimisation for a range of different hardware constraints and got very similar optimised sequence patterns (Supplementary Material Section S1). The main difference we found for different hardware constraints is that the optimal diffusion time and gradient duration both reduce as the maximum gradient strength increases, which is expected as the b-value is kept fixed at *b* = 8 ms/ *μ*m^2^. Furthermore, we found that the TDR values were drastically reduced as the maximum gradient strength is reduced. This, in particularly affects clinical gradient strength values (*G* < 80mT/m) for which TDR was very low of 0.106, and in particular the normalised signal difference itself, S_2_ - S_1,_ was at maximum 0.02, in the same order or less as the noise levels. Connectome scanner constraints on the other hand produced much larger TDR of 0.44 and signal difference S_2_ - S_1_ of 0.08.

#### 3.1.2 Simulation 2: effect of restriction size on TDR

This section explores the TDR contrast over a wide range of pore size distributions, to explore the types of structures visible through TDR. Following the previous results which show that the optimised sequences are very similar across a range of small and large cylinders and spheres, in this simulation we chose the optimised parameters obtained for the large cylinder distribution, thus for S_1_ we choose Δ= 8.9 ms, *δ* = 6.9 ms and for S_2_ we choose Δ= 31 ms, *δ* = 14.08 ms.

**Fig. 4:**
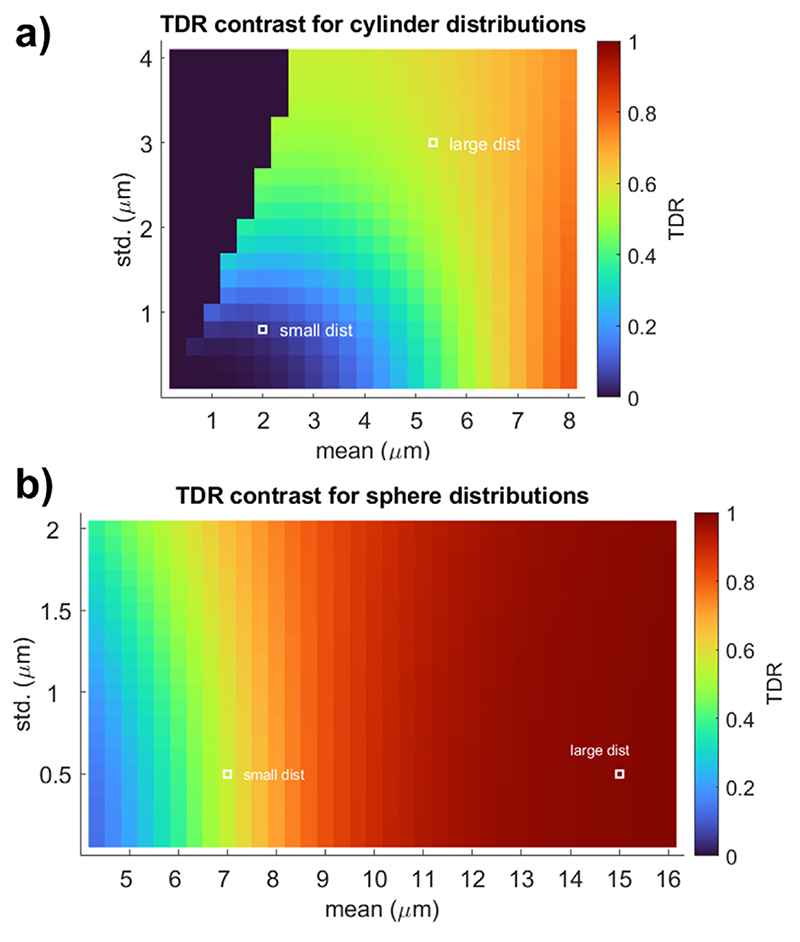
Noise-free TDR values calculated for sequences with *b* = 8 ms/*μ*m^2^ optimised parameters for G_max_ = 600 mT/m: S_1_: Δ = 8.9 ms, *δ* = 6.7 ms; S_2o_: Δ = 30.9 ms, *δ* = 14.1 ms. The signal is simulated across a wide range of (a) cylinder and (b) spherical diameter distributions. For cylinders, we simulate Gamma distributions, and for spheres we simulate Gaussian distributions, which reflect the size distributions usually measured in the tissue. The typical large and small size distributions presented in Fig. 2 are shown using white markers. For cylinders, the gamma distributions are truncated at 20 *μ* m to match realistic values from the tissue; thus, combinations of parameters where this truncation changes the mean by more than 10% were not considered. Equivalent figure for *b* = 20 ms/ *μ*m^2^ is Fig. S3 presented in Supplementary Material.

Fig. 4a illustrates TDR values across substrates consisting of cylinder distribution with a wide range of parameters (mean along the x-axis and standard deviation along the y-axis), calculated based on the optimised sequences above. The plot presents the overall link between the axon distribution sizes and TDR and shows that distributions with the mean below 3 *μ*m and standard deviation below 1 *μ*m - resolution limit for this gradient strength - have TDR values close to zero, and hence would not be detectable in the images. On the other hand larger axons have sufficiently large TDRs (TDR = 1 is a maximum value) which the approach will pick up, and so will stand out in the TDR map images. This plot shows where typical axon distributions found in the tissue would be: “small dist” matches a previous model of callosal white matter (Grussu et al., 2019) which is undetectable with this approach (as expected based on the max gradient strength used (Nilsson et al., 2017)) and “large dist” replicates the white matter structure in the spinal cord, which is within the sensitivity of the TDR approach.

The isocolors in Fig. 4a show existing ambiguities in associating a specific TDR value to a single size distribution. Multiple size distributions can provide the same TDR value: e.g., all the distributions characterised by mean and standard deviation along the green area in Fig. 4 would all contribute to a TDR value of ~0.4. This is expected to be seen in the TDR approach whose main aim is to visualise areas with large, detectable restrictions rather than to map their exact size.

Fig. 4b) shows results for spherical substrates. These are much larger as they represent the structures in the grey matter and their TDR values are much higher and, depending on the SNR, would be detectable in the TDR maps for the hardware parameters selected.

For *b* = 20 ms/ *μ*m^2^ the TDR contrast is very similar to what we discussed above for *b* = 8 ms/ *μ*m^2^, with a difference that the TDR values are in general larger for *b* = 20 ms/ *μ*m^2^ - except for very large cylinders when they are somewhat smaller. Results are presented in Figure S3 in Supplementary Material.

#### 3.1.3 Simulation 3: effect of gradient directions and noise on TDR

**Fig. 5:**
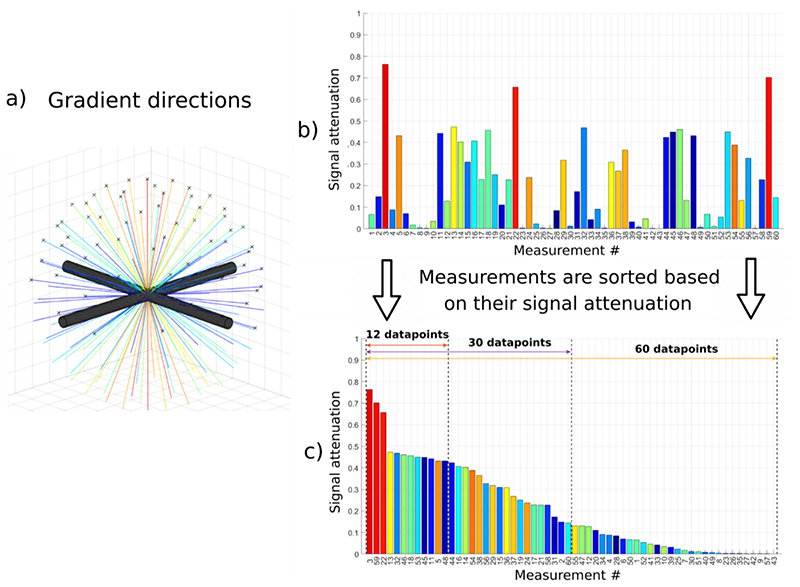
Effect of the gradient direction on the signal coming from two crossing bundles of parallel fibres. a) Gradient directions for an acquisition protocol with 60 measurements. b) Signal attenuation for each diffusion direction simulated for a substrate consisting of two perpendicular fibres / bundles each with Gamma diameter distributions of mean = 5.33 *μ*m, std = 3.00 *μ*m (the same as used in the ‘large axon’ simulations). c) Signal attenuations sorted in descending order and examples of subsets with different numbers of measurements.

It is well known that when imaging a single bundle of parallel fibres, the intra-axonal signal obtained depends on the angle between the orientation of the fibre bundle and the direction of the diffusion sensitising gradients: orthogonal gradients produce small signal attenuation (and thus higher overall diffusion-weighted signal), whilst parallel gradients create stronger signal attenuation and return lower signal. This effect generally increases with b-value (Drobnjak et al., 2016) and would be strongly emphasised in the case of the TDR approach. A similar rationale applies to imaging more than one fibre bundle: the gradient directions returning the highest signals are those which are close to perpendicular to one or more fibre bundles; meanwhile gradient directions which are not close to perpendicular to any of the fibre bundles show very low signal. This concept is represented in Fig. 5, which shows signal measurements for two crossing fibre bundles and 60 uniformly distributed gradient directions (Fig. 5a). Fig. 5b shows all the 60 signal measurements colour coded according to the angle between the plane of the fibres and the gradient direction shown in a) (e.g. red are gradient directions most perpendicular to the fibres). Fig. 5c shows all 60 signal measurements ordered from the highest to the lowest signal to emphasise the impact that gradient direction has on the signal itself. It can be seen how the signal measurements are highly distributed from very high, through medium intensity and finally some measurements with very low signal.

In order to investigate whether this effect affects TDR, we divide the signal measurements into subsets and evaluate TDR for each different subset. The idea is to explore whether using a subset of gradient directions that creates the highest signal is more optimal (i.e. maximises TDR) compared to when using the full set of 60 uniformly distributed directions which includes both the high and the low signals. Note that the data are already measured for all 60 uniformly distributed gradient directions, and this subset selection is happening at the post-processing stage, once the signal intensities have been ordered.

We use the definition of TDR outlined in Section 2.1. and calculate it for different subsets using the following equation:

$$TDR_{M}=\frac{\sum_{j=1}^{M}S_{2,j}-\sum_{j=1}^{M}S_{1,j}}{\sum_{j=1}^{M}S_{2,j}}$$

where M is the number of gradient directions in the subset (N corresponds to the full set of uniformly distributed directions and 1 ≤ *M* ≤ *N*) and *j* = 1..M is the index of the ordered signal measurements from the highest intensity to the lowest intensity (as shown in Fig. 5c). The ordering is performed on the average of the two signals (*S*_1_ + *S*_2_)/2, in order to reduce the effect of noise on the sorting process. In our simulations we used HARDI acquisitions of 60 uniformly distributed gradient directions (determined by electrostatic repulsion), hence *N* = 60 and *T DR*_60_ is equivalent to the full original formulation of TDR (ie. signal S_1_ and S_2_ are averaged across all 60 gradient directions acquired). To illustrate this further: *T DR*_1_ is calculated using only one gradient direction - the one that provides maximum signal intensity; *T DR*_2_ uses the two strongest signal measurements to calculate the average, the one used in *T DR*_1_ and another one in the ordering etc.

**Fig. 6:**
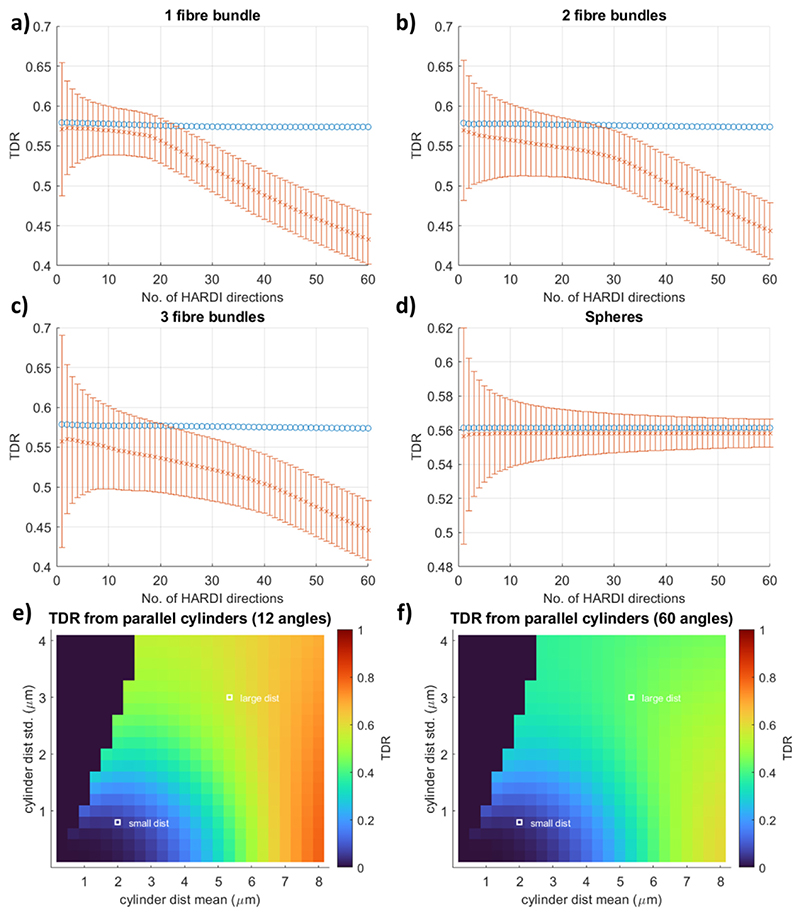
TDR values for noise free (blue circles, SNR = inf) and noisy (orange crosses, Rician noise with SNR = 20) signals as a function of the number of gradient directions included in the analysis in different substrates: (a–c) bundles of cylinders with one, two or three fibre orientations crossing at 90°. All bundles have a Gamma distribution of sizes with mean = 5.33 *μ*m and std = 3.00 *μ*m; (d) a Gaussian distribution of spheres with mean = 7 *μ*m and std = 0.5 *μ*m. The orange error bars show 1 standard deviation of the estimated TDR over 100,000 noise instances. As illustrated in (a–c), for the different fibre configurations, TDR values estimated from noisy data are below the expected noise-free values. All simulations are performed using the optimised sequence parameters for large cylinders. Equivalent figure for *b* = 20 ms/ *μ*m^2^ is Fig. S4 presented in Supplementary Material.

The results for TDR calculated for every subset from *T DR*_1_ to *T DR*_60_ are provided in Fig. 6(a–d). Fig. 6a shows the TDR values for a substrate consisting of one fibre bundle of parallel cylinders. Results are presented both for the noise-free scenario (blue circles) and the scenario where Rician noise (SNR = 20) has been added (orange error bars present the standard deviation over the noise instances). In the scenario without noise, TDR remains equal to the ground truth regardless of how many gradient directions are used in the TDR calculation. However, once simulated Rician noise is added to the signals, due to the Rician floor effect, the results show a decrease in TDR and a reduction in the accuracy of the calculated TDR value as the number of gradient directions included in the calculation increases. In contrast, the precision of the calculation is improved with more measurements and hence for this substrate with one fibre bundle, using *T DR*_12_ which corresponds to the subset of ~20% of ordered gradient directions that provide the highest signal values, yields an optimal balance between accuracy and precision of TDR estimates.

Figs. 6b and c show that similar effects occur for substrates consisting of two and three fibre bundles (fibre bundles are mutually perpendicular to allow for testing of the most extreme cases). The accuracy of estimated TDR lowers as the number of fibre bundles increases, however the trend that the accuracy reduces with increasing the size of the ordered subset is equally present suggesting that optimisation in the presence of Rician noise would be very beneficial and would provide higher TDR contrast.

For a substrate consisting of spheres (Fig. 6d) the results are very different. Accuracy is pretty stable regardless of the number of signal measurements used for both the no-noise and noisy case. This is as expected since signal from spherical substrates is indifferent to gradient direction as opposed to the fibres which are highly sensitive to it. Similarly to the fibre simulations, the precision is improved with the number of measurements and hence the optimal solution for spherical substrates is that which maximises the number of measurements - i.e. the full set of HARDI acquisitions (*T DR*_60_ for our simulations). Hence, for isotropic pores, as well as uniformly orientated fibres (data not shown), using more gradient directions is optimal, with TDR values remaining accurate even when all directions are used.

We also note that the estimated TDR values for the substrates with one, two or three fibre bundles should ideally be the same, as the size distributions of the substrate cylinders are the same regardless of the fibre orientation distribution. When calculating the coefficient of variation of TDR values across the three substrates, for an SNR of 20 when 60 HARDI gradient directions have been acquired, we see a minimum for subsets with 27/60 gradient directions, as illustrated in Figure S6 in Supplementary Information. For other SNR levels and numbers of acquired gradient directions, the exact number of measurements in the optimal subsets might vary slightly, nevertheless, using a smaller subset of gradient directions appears optimal, also for SNR = 50 and 30 measured gradient directions, as illustrated in Figures S5-S6 in Supplementary Information. Overall, in all fibre scenarios considered, using ~50% of the gradient directions acquired provides a more accurate TDR estimate than when all gradient directions are used; in the SNR 50 scenarios or when 60 HARDI gradient directions are acquired, using ~33–50% of the directions also reduces the coefficient of variation between the three fibre scenarios compared to when all gradient directions are used.

Figs. 6e and f show *T DR*_12_ and *T DR*_60_, respectively, in the presence of noise, for the single fibre bundle scenario, across a wide range of different cylinder distributions. We can see that using all 60 directions results in the reduction of TDR compared to the ground truth (no-noise case, Fig. 4a) for many large distributions: this also reduces the difference in TDR between large and small distributions, reducing the potential contrast of a TDR image. *T DR*_12_ is on the other hand considerably higher and closer to the ground truth: for example for the distribution of cylinders with a mean 5.33 *μ*m, *T DR*_12_ is 1.6% lower compared to the ground truth while *T DR*_60_, is 24.5% lower - a greater than 15 times difference in percentage error - which in the real-world scenario could create contrast that is not sufficiently detectable.

For *b* = 20 ms/ *μ*m^2^ the results are very similar to what we discussed above for *b* = 8 ms/ *μ*m^2^. The main difference is that for cylindrical substrates, especially those with larger cylinders, decrease in accuracy is even more pronounced, and the optimal subset size for calculating TDR is smaller. For spherical substrates the accuracy is the same however the precision is, as expected, much improved. Results are presented in Figure S4 in Supplementary Material.

### 3.2 Preclinical experiments

#### 3.2.1 Optimised and non-optimised acquisition protocols

Diffusion MRI measurements for TDR contrast in ex-vivo spinal cord were acquired following the non-optimised TDR approach, where the diffusion time is varied between the two shells, as well the optimised gradient waveforms proposed in this work. The measurements were repeated for a maximum gradient strength of 600 mT/m, a value widely available on pre-clinical systems as well as 2500 mT/m that is available on typical microimaging probes. The specific values for the non-optimised and optimised protocols are given below:

Non-optimised protocol:

- Shell 1 consists of waveforms with short gradient duration and short diffusion time:
  ○ *b* = 8 ms/*μ*m^2^, G_max_ = 600 mT/m: *δ* = 6.9 ms, Δ = 9 ms;
  ○ *b* = 8 ms/*μ*m2, Gmax = 2500 mT/m: *δ* = 2.2 ms, Δ = 4.5 ms.
  ○ *b* = 20 ms/*μ*m2, Gmax = 600 mT/m: *δ* = 9.7 ms, Δ = 11.7 ms;
  ○ *b* = 20 ms/*μ*m2, Gmax = 2500 mT/m: *δ* = 3.2 ms, Δ = 5.2 ms.
- The optimal values from simulations were slightly adjusted to accommodate scanner constraints such as finite slew rates. This shell is used both for the original and the optimised TDR calculation, as prescribed by the simulation results.
- Shell 2n consists of waveforms with short gradient duration and long diffusion time:
  ○ *b* = 8 ms/ *μ*m^2^, G_max_ = 600 mT/m: *δ* = 6.9 ms, Δ= 34.6 ms;
  ○ *b* = 8 ms/ *μ*m^2^, G_max_ = 2500 mT/m: *δ* = 2.2 ms, Δ= 39 ms.
  ○ *b* = 20 ms/ *μ*m^2^, G_max_ = 600 mT/m: *δ* = 9.7 ms, Δ= 32 ms;
  ○ *b* = 20 ms/ *μ*m^2^, G_max_ = 2500 mT/m: *δ* = 3.2 ms, Δ= 38.5 ms.

The maximum diffusion time given the same echo time was chosen. TDR values computed from Shell 1 and Shell 2n are referred to as non-optimised.

Optimised protocol:

- Shell 1 is the same as for the non-optimised protocol.
- Shell 2o consists of waveforms with long gradient duration and long diffusion time:
  ○ *b* = 8 ms/ *μ*m2, Gmax = 600 mT/m: *δ* = 14 ms, Δ= 27.5 ms;
  ○ *b* = 8 ms/ *μ*m2, Gmax = 2500 mT/m: *δ* = 15 ms, Δ= 26.5 ms.
  ○ *b* = 20 ms/ *μ*m2, Gmax = 600 mT/m: *δ* = 15 ms, Δ= 26.5 ms;
  ○ *b* = 20 ms/ *μ*m2, Gmax = 2500 mT/m: *δ* = 15 ms, Δ= 26.5 ms.

The timing parameters are adapted from the numerical optimization. TDR values computed from Shell 1 and Shell 2o are referred to as optimised.

**Fig. 7:**
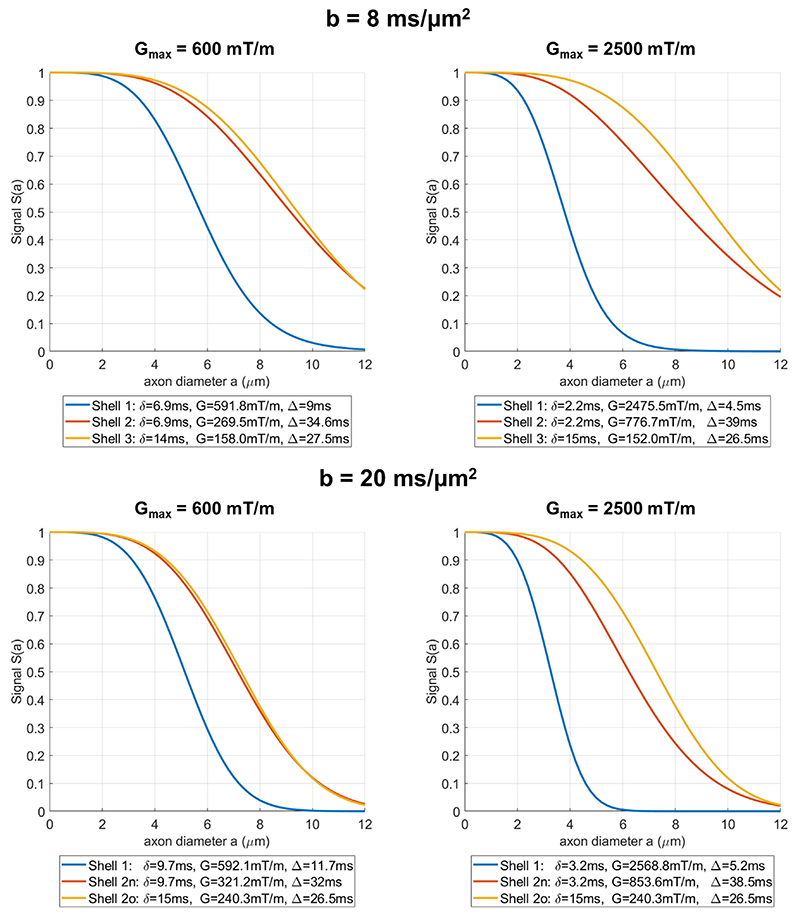
Simulations for signal attenuation profiles as a function of cylinder diameter for the three shells used in the pre-clinical acquisition at *b* = 8 ms/*μ*m^2^ and *b* = 20 ms/*μ*m^2^, both for G_max_ = 600 mT/m (left) and G_max_ = 2500 mT/m. The signals were simulated for single cylinders and diffusion gradients perpendicular to the fibre with intrinsic diffusivity of *D* = 2 *μ*m^2^/ms.

The signal attenuation profiles for these gradient waveforms plotted against axon diameter are presented in Fig. 7, illustrating that indeed the difference between Shell 2o and Shell 1 (optimised) is larger than between Shell 2n and Shell 1 (not-optimised).

##### TDR contrast in spinal cord

**Fig. 8:**
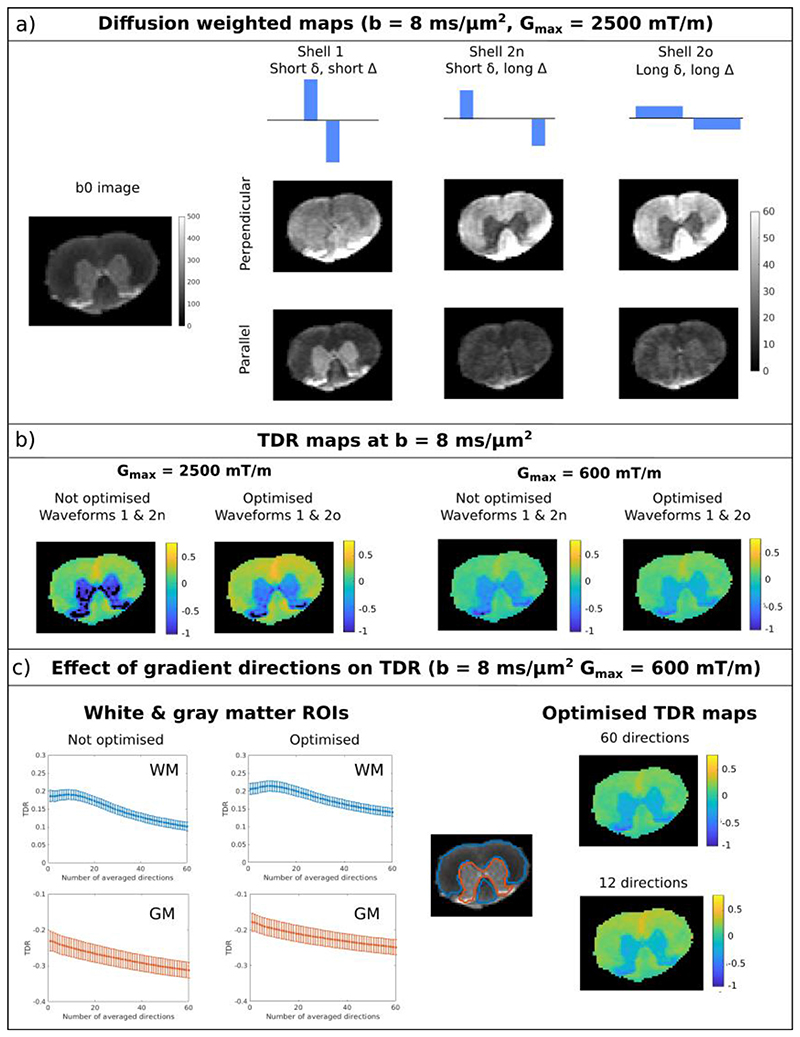
Optimisation of TDR acquisition including waveforms (a-b) and number of gradient directions (b). a) left: T2 weighted image of the rat spinal cord without diffusion gradients. White matter and grey matter regions are delineated with blue and orange contours, respectively. Right: schematic depiction of the gradient waveforms for the three shells (top) and the corresponding diffusion weighted maps for a perpendicular (middle) and a parallel (bottom) gradient direction. The maps are shown for denoised data. b) TDR maps for maximum gradient strength of 600 and 2500 mT/m for optimised and non-optimised gradient waveforms. c) left: TDR values as a function of how many gradient directions were included in the signal average for white matter (blue) and grey matter (orange) ROIs. right: TDR maps for measurement subsets with 12/60 and 60/60 directions. Equivalent figure for *b* = 20 ms/*μ*m^2^ is Fig. S7 presented in Supplementary Material.

Fig. 8a illustrates the data acquired in the ex-vivo rat spinal cord for *b* = 8 ms/ *μ*m^2^, where WM and GM regions are outlined on the b0 image. The normalised diffusion weighted maps are shown for the three different waveforms when the gradient is either close to parallel or perpendicular to the spinal cord fibres. Indeed, there is a pronounced change in contrast between the different shells. For the perpendicular direction, the signal in white matter increases between shell 1 and shells 2n & 2o, while the signal in grey matter decreases. For the parallel direction, the change is less pronounced in white matter, nevertheless there is still a pronounced decrease in grey matter. Estimated SNR values in white and grey matter were 78 ± 28 and 148 ± 62, respectively.

Fig. 8b presents TDR maps calculated based on all gradient directions for the two scenarios with *b* = 8 ms/ *μ*m^2^ G_max_ = 600 mT/m as well as for G_max_ = 2500 mT/m. In spinal cord white matter, TDR contrast is positive. For G_max_ = 600 mT/m, optimised TDR values in WM are between 0 and 0.4, matching a wide range of axonal distributions presented in Fig. 4a for the same gradient strength. On the other hand, in grey matter, TDR values are negative, showing that a model of pure restriction (either in cylinders and/or spheres) does not accurately represent the diffusion time dependence in the tissue.

For both gradient strengths, we see that the TDR contrast provided by the optimised pair of gradient waveforms (i.e. Shell 1 and Shell 2o) is higher than the values from the non-optimised pair (Shell 1 and Shell 2n). Moreover, TDR values obtained with stronger gradients are higher, matching the simulation results presented in Supplementary Information in Figure S1, as well as previous results regarding estimating axon diameters (Fan et al., 2020; Drobnjak et al., 2016; Nilsson et al., 2017).

Fig. 8c illustrates the effect of using a subset of gradient directions in the computation of TDR. To assess the effect of gradient direction on the TDR estimate in the spinal cord, where white matter tracts are highly anisotropic, the gradient directions were voxelwise sorted based on the average signal intensity in the three shells. Then, TDR was computed from subsets of gradient orientations with an increasing number of directions, as detailed in Fig. 5. We find that for WM, both the optimised and non-optimised TDR values decrease as more gradient directions are included in the signal average. Including only ~ 20% of the data points (i.e. 12/60 directions) already provides a good balance towards maximising TDR while minimising the effect of noise, corroborating the simulation results. In GM, there is also a dependence of TDR on the number of measurements, likely due to the directionality of the neurites (Grussu et al., 2017), and we estimated negative TDR values. Similar results are observed for the second spinal cord segment.

Overall, for a given gradient strength and echo time, the highest TDR contrast in spinal cord white matter is obtained using the optimised pair of gradient waveforms and approx 12/60 directions from a fully acquired HARDI shell. (Dula et al., 2010)

Figure S7 in the Supplementary Material shows equivalent results but for *b* = 20 ms/ *μ*m^2^. The TDR maps and the overall results are very similar between the two b-values in line with simulations presented in Section 3.1. There is a small difference in the effect of the number of gradient directions - in WM for the larger b-value the accuracy decreases faster as the number of gradient directions increases. This is also consistent with simulation results presented in Section 3.1.

**Fig. 9:**
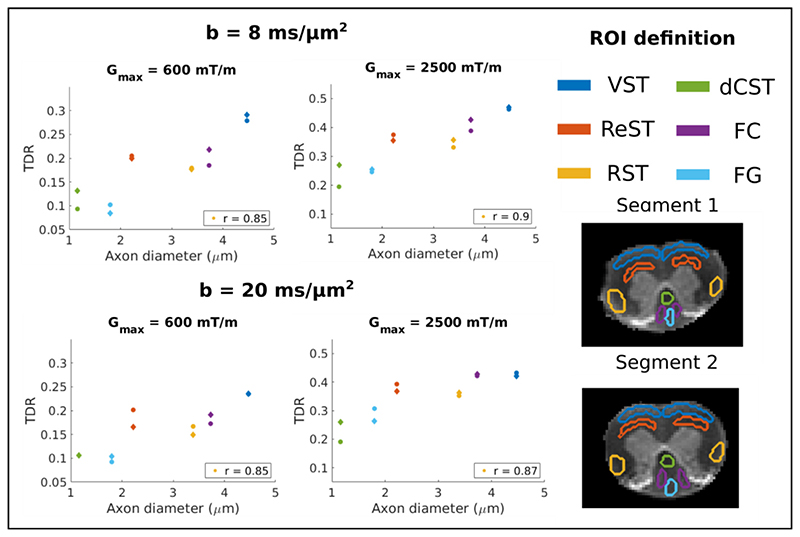
Comparison between TDR values and axon diameters reported in the literature in 6 spinal cord WM ROIs (Vestibulospinal (VST) - blue, Reticulospinal (ReST) - orange, Rubrospinal (RST) - yellow, dorsal corticospinal (dCST) - green, Funiculus Cuneatus (FC) - purple and Funiculus Gracilis (FG) - light blue). The ROIs are manually delineated and colour coded as illustrated on the left. Each segment is represented with a different marker shape in the correlation plot. Strong correlations between mean TDR values and axon diameter are observed for all the analysed sequences, both for Gmax = 600 mT/m, with a correlation coefficient of 0.85 (*p* < 0.01) for both b-values, as well as for Gmax = 2500 mT/m with a correlation coefficient of 0.9 (*p* < 0.01) for *b* = 8 ms/*μ*m^2^ and 0.87 (*p* < 0.01) for *b* = 20 ms/*μ*m^2^.

Fig. 9 compares TDR with axon diameter values previously reported in 6 different WM ROIs. The TDR values are calculated voxelwise from the optimised waveforms using a subset of 12 gradient directions, to maximise the contrast. Then, the mean TDR value in each ROI is computed. Strong correlations between mean TDR values and axon diameter are observed for all the analysed sequences, both for G_max_ = 600 mT/m, with a correlation coefficient of 0.85 (*p* < 0.01) for both b-values, as well as for G_max_ = 2500 mT/m with a correlation coefficient of 0.9 (*p* < 0.01) for *b* = 8 ms/ *μ*m^2^ and 0.87 (*p* < 0.01) for *b* = 20 ms/ *μ*m^2^. The TDR values are also similar for the two different b-values. Moreover, the TDR values for the two spinal cord segments, that were mounted and imaged separately, are highly consistent.

### 3.3 Effect of extra-axonal space and orientation dispersion

Monte Carlo simulations with substrates consisting of parallel cylinders with an intra-axonal fraction of 0.6 were employed to assess the effect of extra-axonal space.

**Fig. 10:**
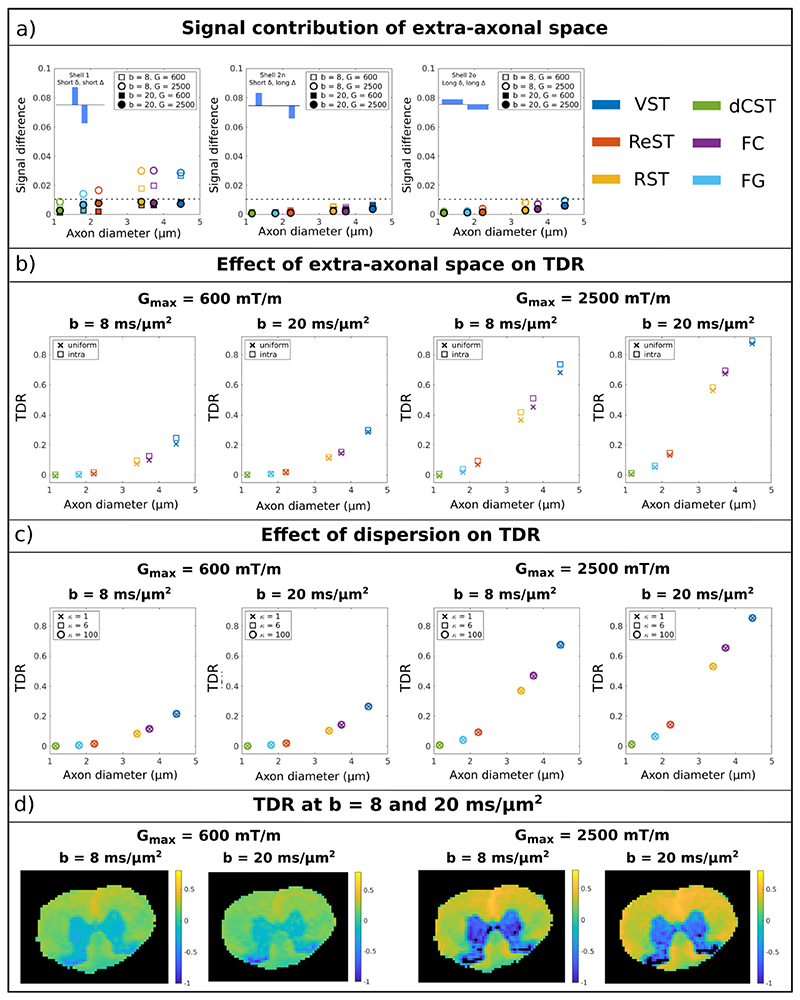
(a) Signal contribution of extra-axonal space calculated from Monte Carlo simulations of parallel cylinders with a Gamma distribution of radii corresponding to the spinal cord ROI values. The signal is the average over the 12 gradient directions used for computing TDR_12_. The contributions are shown for the different waveforms (left to right), as well as for different b-values and maximum gradient strengths (markers). The largest differences observed for *b* = 8 ms/*μ*m^2^ do not exceed 0.03 for the simulated substrates. The dotted line represents 0.01 signal difference. b) Optimised TDR (12 gradient directions, optimised waveforms) as a function of axon diameter for different protocols, when particles in the MC simulation are distributed either uniformly (‘x’) or just inside the cylinders (‘□’). c) Optimised intra-axonal TDR (12 gradient directions, optimised waveforms) as a function of axon diameter for dispersed cylinders following a Watson distribution with different concentration parameters *κ* = {1, 6, 100}. The TDR values overlap for the different *κ* values. c) Comparison of optimised TDR for the data acquired with *b* = 20 ms/ *μ*m^2^ and 8 ms/ *μ*m^2^ for the two gradient strengths.

Fig. 10a presents the signal contribution of the extra-axonal space, i.e. S_total_-S_intra_, as a function of mean axon diameter for the three waveforms and different combinations of b-value and maximum gradient strength. The shown signal is the average over the first 12 directions, that will later be used to compute the optimised TDR. In most cases the signal contribution of the extra-axonal space is less than 0.01 (on a scale from 0 to 1). For sequences with short diffusion time, *b* = 8 ms/ *μ*m^2^ and G_max_ = 2500 mT/m the differences are slightly larger, up to 0.03, for diameters > 3 *μ*m.

Fig. 10b shows the effect of the extra-axonal signal propagated to the TDR values calculated from 12 directions and the optimised waveforms. The TDR values calculated from simulations with a uniform distribution of spins (i.e. including both intra and extra-axonal space) are lower than the values simulated only with an intra-axonal spin distribution. The maximum differences for the various sequences are: 0.041 (16%) and 0.053 (7.5%) for *b* = 8 ms/ *μ*m^2^ with G_max_ = 600 mT/m and 2500 mT/m, respectively, and 0.011 (3.8%) and 0.022 (2.4%) for *b* = 20 ms/ *μ*m^2^ with G_max_ = 600 mT/m and 2500 mT/m, respectively. Similarly to other results throughout the paper we see that the TDR is higher for G_max_ = 2500mT/m compared to 600mT/m. Finally, as expected from results presented in Fig. 4 and Figure S3, the TDR is slightly different between two different b-values. For the substrates considered here, TDR for *b* = 20 ms/ *μ*m^2^ is slightly higher, with differences up to 0.05 for G_max_ = 600 mT/m and 0.017 for Gmax = 2500 mT/m, respectively.

Fig. 10c illustrates the effect of fibre dispersion on the optimised TDR values (12 directions, optimised waveform) based on numerical simulations performed in MISST for cylinders following a Watson distribution with different concentration parameters. Without noise, TDR values are similar for dispersed fibres, although in the presence of noise orientation dispersion tends to slightly lower the TDR value, especially for sequences with *b* = 20 ms/ *μ*m^2^ and the stronger maximum gradient (data not shown).

To investigate the effect of b-value on TDR in practice, Fig. 10d compares the experimental TDR values calculated at *b* = 8 ms/ *μ*m^2^ and 20 ms/ *μ*m^2^ in the spinal cord for the two maximum gradients. The average TDR values in WM are similar for the two b-values, as follows: for G_max_ = 600 mT/m, TDR = 0.21 ± 0.07 and 0.18 ± 0.06 for *b* = 8 ms/ *μ*m^2^ and *b* = 20 ms/ *μ*m^2^, respectively; for G_max_ = 2500 mT/m, TDR = 0.37 ± 0.09 and 0.37 ± 0.05 for *b* = 20 ms/ *μ*m^2^ and *b* = 8 ms/ *μ*m^2^, respectively.

## 4 Discussion

This work employs numerical simulations to optimise the dMRI acquisition for maximal TDR contrast. The simulation results are validated by ex-vivo diffusion MRI data from both WM and GM tissues in rat spinal cord.

### 4.1 TDR contrast and waveform optimisation

Through our investigation of pulse sequence shapes for standard single diffusion encoding experiments, we find that optimised TDR requires short-*δ*, high-G sequences contrasted with long-*δ*, low-G sequences. These results are consistent for a wide range of substrates (e.g. restricted diffusion in cylinders and spheres with different size distributions) and sequence constraints (maximum gradient strength, total gradient duration, etc.), and are in line with the optimisation results of previous studies (Alexander et al., 2010; Drobnjak et al., 2016; Nilsson et al., 2017; Ianus et al., 2021). Intuitively, this can be understood by the following reasoning. Given the restriction length r, assuming the Gaussian phase approximation and the long-pulses limit for simplicity, the signal attenuation for the SDE sequence is ln S ∝ - r^4^g^2^*δ*, where g is the gradient strength amplitude (Veraart et al., 2020; van Gelderen et al., 1994; Neuman, 1974). If we acquire both the signals S_1_ and S_2_ needed to compute the TDR (see Section 2.1) with the same b value, the condition g^2^*δ*^2^(Δ-*δ*/3)=*const* must be satisfied. In order to maximize TDR contrast given the above conditions, we want maximal attenuation for S_1_ which leads to maximizing *const* / [*δ*(Δ-*δ*/3)], and minimal attenuation for S_2_ which leads to minimizing *const* / [*δ*(Δ-*δ*/3)]. Obviously, this can be achieved by acquiring the signal S_1_ using *δ* and Δ as short as possible (consequently, with stronger g given the condition of constant b value) and the signal S_2_ using *δ* and Δ as long as possible (consequently, with weaker g given the condition of constant b value).

The contrast from the optimised TDR is sensitive to a range of restriction sizes, as illustrated in Fig. 4, and has the potential to distinguish distributions containing large axons from those containing small axons. As expected, size distributions with different combinations of mean and standard deviation can yield very similar TDR values, as illustrated by areas with similar colours in Fig. 4. This effect is more pronounced for distributions with longer tails, such as the gamma distribution used for cylindrical substrates, compared to normal distributions used for spherical substrates, where we observe a better correspondence between the mean of the distribution and TDR values. This known tail-weighting effect is due to the fact that the signal contribution of each cylinder to the total measured signal is volume-weighted (Alexander et al., 2010; Veraart et al., 2020). As reference, for axon diameter distributions typically found in human brain tissue (i.e. <3 *μ*m), TDR contrast is on the order of 6% or less (Dell’Acqua et al., 2019).

As illustrated both through simulations and experiments, the TDR contrast depends on the maximum gradient strength available on the scanner, and is sensitive to axon diameters that are above the resolution limit, i.e. the minimum diameter that can be distinguished (Alexander et al., 2010; Drobnjak et al., 2016; Nilsson et al., 2017). For sizes below the resolution limit, TDR is zero. In regards to clinical imaging and maximum gradient strength needed for TDR mapping, we find that gradient strengths on typical clinical scanners (below 80 mT/m) are not suitable as they do not provide sufficient contrast, and stronger gradients are needed, e.g. such as 200 mT/m or more available on the Connectome scanner (results presented in Supplementary Material Section S1).

### 4.2 Effect of extra-axonal space

The TDR exploits ultra-high b-values to suppress the contribution from water diffusing in the extra-axonal space, and enhance the sensitivity of the dMRI measurements to water diffusing in the restricted intra-cellular space. Previous work demonstrated, by identifying the power-law signature specific of narrow cylinders, that b ≿ 7 ms/ *μ*m^2^ is enough to assume that the observed signal originates from inside the axons only (Veraart et al., 2019). Hence, in this study we investigate sequences at *b* = 8 ms/ *μ*m^2^, which is at the limit of that requirement, as these can be achieved on pre-clinical and high performance clinical scanners for a range of gradient waveforms. We find that for those, the extra-axonal signal is negligible (normalised signal below 0.01) for most configurations (including complex fibre geometries as seen in Supplementary Material Section S5) and protocols (Fig. 10a). Only the largest axonal configurations (*>*3um) for very short diffusion times (~5 ms) reach non-negligible but still very low extra-axonal signal of 0.03. This seems to originate from axonal configurations with geometrical repetitions, inducing slow diffusion or even restriction in some of the extra-axonal space, a feature we expect would be much smaller in the real data.

We found that the impact of this on TDR was not significant. We investigated *b* = 20 ms/ *μ*m, for which the extra-axonal signal is negligible (normalised signal below 0.01) for all axonal configurations and protocols we investigated and TDR values of intra-axonal space almost identical as that of the uniform space (intra+extra-axonal) [Fig. 10b]. When compared in simulations TDR for *b* = 8 ms/ *μ*m^2^ has differences slightly larger for certain axonal configurations and *G* = 2500mT/m but overall still very small. When compared in pre-clinical data, we find that the TDR maps between the two b-values are extremely similar and with matching patterns and values, suggesting that *b* = 8 ms/ *μ*m^2^ is sufficiently large to satisfy the TDR assumptions.

### 4.3 TDR in the presence of noise

In the original TDR, it was proposed to use the direction-averaged signal to remove/mitigate the bias due to orientation dispersion. In this work we show that, when imaging structures consisting of one or several anisotropic fibre bundles (e.g. coherent white matter fibres, but also regions with crossing fibres), in the presence of Rician noise, the TDR contrast can be further improved by using only a subset of the highest-signal gradient directions. This happens because for some gradient directions the measured signal is very low and decays to the noise floor, therefore adding a bias in the powder averaged signal and subsequent TDR calculation. Thus, removing these measurements improves the accuracy of TDR estimation. For relatively coherent fibres, both simulations and experimental results suggest that using approx 20% of the measurements is optimal. For more complex fibre configurations (e.g. two or three crossing fibre bundles), larger subsets are more appropriate, with the exact optimal percentage of gradient directions depending on the SNR as well as the fibre configurations. If we are to choose a single percentage, for example to calculate TDR across the brain where there are voxels with different fibre orientations, our simulations suggest that considering a subset with e.g. ~50% of measurements, improves TDR accuracy and decreases its variance across fibre orientations compared to using the entire dataset. Nevertheless, the optimal percentage should be decided based on the SNR of the data, the number of HARDI directions and expected fibre orientations in the sample.

This bias in TDR can also be mitigated by employing real valued data (Eichner et al., 2015) instead of magnitude data and removing the effect of the Rician noise floor. Indeed, simulations employing Gaussian noise result in similar TDR values for different numbers of gradient directions (data not shown).

### 4.4 TDR in ex-vivo spinal cord: white matter

The TDR contrast in the ex-vivo rat spinal cord closely follows the predictions from simulations, with higher values for the optimised waveforms and ~20% of the directions. We have also observed very strong correlations between TDR and axon diameter values reported in the literature in different ROIs, with correlation coefficients above 0.85 both for a weaker gradient (600 mT/m) and a stronger gradient (2500 mT/m). As discussed above, TDR is sensitive to the volume weighted distribution of axons, and therefore the mean diameter calculated from electron microscopy might not be the best quantity for comparison, nevertheless, there is a clear trend, as illustrated in Fig. 9. The trends of TDR are also consistent with previous MRI correlates of axon diameter, both using diffusion data as well as relaxometry (Shemesh, 2018; Anaby et al., 2019; Fick et al., 2017; Nunes et al., 2017).

The curves presented in Fig. 1 show a slightly different dependence of TDR on axon diameter compared to the trends shown in Fig. 9- namely that in simulations the TDR curve goes to zero for small axons and in fixed rat spinal cord it does not. Indeed, when considering straight cylinders (Fig. 10), simulated TDR values for axons below 2 um are close to zero, while TDR values measured in the spinal cord are larger (~0.1 for Gmax = 600 and 0.2 for Gmax = 2500). Nevertheless, the TDR values simulated in the more realistic substrates with undulation and varying axon diameter indicate higher values compared to straight cylinders, that are similar to the ones measured in the spinal cord in ROIs known to have similar axon sizes, see Table S1 in Supplementary material. Since TDR is not aiming to measure pore sizes quantitatively, these differences do not affect the interpretation of the results significantly.

### 4.5 TDR in ex vivo spinal cord: grey matter

In *ex vivo* spinal cord grey matter we measure negative TDR values. These results are not consistent with simulations of TDR when diffusion is influenced by restricting barriers in different scenarios: i) totally restricted diffusion in spheres and/or cylinders, ii) randomly packed parallel cylinders with a uniform distribution of spins, iii) packed complex fibre geometries featuring orientation dispersion, undulation and varying diameter along the fibre (see section S6 in SI) with a uniform distribution of spins. This suggests that effects, other than pure restriction, influence the diffusion time dependence in grey matter. These results show for the first time that for measurements with high diffusion weighting (i.e. ≥8 ms/ *μ*m^2^), the signal is showing a pronounced decrease with increasing diffusion time in spinal cord grey matter. This is consistent with recent preclinical studies focusing on brain grey matter, suggesting that inter-compartmental exchange is a highly plausible explanation for the diffusion time dependence, both *in vivo* and *ex vivo* (Jelescu et al., 2022; Olesen et al., 2022; Ianus et al., 2022). In this case, TDR may reflect exchange between neurites and extra-cellular space, between soma and extra-cellular space and/or between soma and neurites (Ianus et al., 2021; Jelescu et al., 2022; Olesen et al., 2022). Since TDR was originally proposed to provide information about restriction effects in the WM, here we focus our optimization and investigation on WM. The observed discrepancy between simulated and measured TDR values in the GM are of great interest, but require further dedicated investigations, which goes beyond the scope of this work.

### 4.6 Going beyond single diffusion encoding

Here we chose to look at only SDE sequences since this is the first paper to look into TDR following the preliminary results presented in Dell’Acqua, proc. ISMRM 2019) (Dell’Acqua et al., 2019) and we felt that starting from the most standard sequence and exploring it at depth (both in simulation and pre-clinically) was sensible. We showed that the TDR can work well already with SDE, however the TDR contrast could be further improved by including other waveforms in the optimization, for example oscillating gradients (Drobnjak et al., 2016), and a similar approach has been explored recently in simulations using such waveforms (Harkins et al., 2021). If high frequency oscillating gradients cannot usually achieve the desired high b-values for a practical echo time, low frequency oscillations could potentially improve TDR contrast, as they were shown in the past to increase the sensitivity towards small axon diameters. Nevertheless, these sequences are not widely available, neither on clinical nor on pre-clinical scanners, therefore here we focused on standard single diffusion encoding acquisitions. For larger restriction sizes, using SDE sequences in a stimulated echo sequence rather than the standard spin-echo preparation might be beneficial as it allows to reach larger gradient separations, a period where the signal is governed by a slower T1 decay rather than the faster T2 decay.

### 4.7 Limitations

This work optimises TDR based on simple and ideal geometries (parallel cylinders and spheres), nevertheless, the results are corroborated for WM (where TDR works as expected) using both real data from spinal cord and simulations performed for more complex substrates including orientation dispersion, extra-axonal space and realistic axonal shapes. However, none of these substrates consider exchange between compartments, so a full exploration of negative TDR values in GM is not possible. The future exploration of complex substrates with a wider range of parameters describing dispersion, axon curvature (Nilsson et al., 2012; Lee et al., 2020), beads (Alves et al., 2022; Budde and Frank, 2010), and/or exchange might provide more insight into the behaviour of TDR across various possible microstructures. Another simulation limitation is that noise is simulated using the Rician distribution; strictly speaking, noise across a multi-channel receive coil (e.g., 32 channels for many clinical scanners) should have a non-central Chi distribution. Moreover, TDR contrast could be further improved by considering other gradient shapes, such as oscillating gradients or spin preparations, e.g. stimulated echo, nevertheless, this would require the use of new sequences that are not widely available at the moment. Finally, in our simulations we assume infinite slew rates in the sequence, which although are a very good approximation for the pre-clinical scanners (the scanner we use has a slew rate of 25,000 mT/m) are not so well suited for clinical scanners. Hence we tested the finite slew rate of 200 mT/m (typical of Connectome Scanner) that can accommodate the physiological constraints and found that this has not affected the optimisation results significantly (results in the Supplementary Material S2).

We did not estimate the diffusivity directly from our mouse tissue sample: the spinal cord segments did not have clear CSF-containing areas, and they were immersed in fluorinert, which is not MR visible. That said, we have checked the MD values in CSF from previously published mouse brain data acquired on the same system as ours, at the same temperature and following the same perfusion protocol: they were ~2.8 *μ*m^2^/ms and it is very likely that our sample was in line with this.

TDR requires special care when interpreting the results. As TDR is a single-dimensional measure of restriction size, different size distributions can return identical TDR values. Examples of these distributions can be determined through examination of the isocolours in Fig. 4. More generally, when moving beyond substrates consisting simply of cylinders (e.g. as a model of axons in WM) or spheres, there will be a range of microstructure compositions which we can expect to return similar values of TDR.

TDR is also affected by the inherent sensitivity of the acquisition and the diffusion signal to the pore sizes. As shown in previous work (Drobnjak et al., 2016; Nilsson et al., 2017), the sensitivity of the diffusion signal to axon diameter strongly depends on the gradient strength and SNR. These can be used to calculate a size resolution limit below which the signal has minimal sensitivity. While its dependence on the resolution limit is very similar to model-based approaches, TDR has an advantage that it does not try to fit but rather just maps areas where the voxels are more likely to contain pores of sizes above the resolution limit. Hence, its robustness, sensitivity and interpretation will be much improved and more appropriate for some applications compared to the model-based approaches. However, it is also important to highlight that TDR values can be biased by properties of the tissue other than pore size, such as inter-compartment exchange (compartment-specific T2 and T1 relaxation should not bias to TDR values as long as high enough b values are used to suppress extra-cellular signal and TE/TR are kept the same for all the acquisitions). This could be particularly problematic in diseases, where for example demyelination and accumulation of reactive glia may change the exchange properties of the tissue, complicating the interpretation of the results. Future studies investigating TDR sensitivity and specificity to changes in tissue pore sizes in disease might shed a light on the impact of said confounders.

## 5 Conclusion

This work employs simulations and pre-clinical data to show the potential of TDR contrast to characterise restricted diffusion for a wide range of tissue microstructures featuring cylindrical and/or spherical size distributions. Importantly, the TDR contrast can be enhanced by using optimised gradient waveforms, contrasting a short *δ* + high G pulse and a long *δ* + low G pulse, as well as a subset of gradient directions from the acquired shells. In ex-vivo experiments on rat spinal cord, TDR can successfully characterise spinal cord white matter microstructure, showing a strong correlation with axon diameter values from quantitative histology. Overall these results show that the recently proposed TDR approach has a great potential and is a very promising alternative (or potentially complement) to model-based approaches for **informing on** pore sizes in tissue.

## Supplementary Material

Appendix 1

## Data Availability

Simulations are generated using the Microstructure Imaging Sequence Simulation Toolbox (MISST, version 0.93)60, a diffusion MRI simulator that calculates the signal attenuation for various restricted geometries and different gradient waveforms using the matrix formalism61–63. Code for MISST is available at http://mig.cs.ucl.ac.uk/index.php? *n*=Tutorial.MISST. Code written by the authors will be made available before the final submission. The data and code from the preclinical experiments are available at https://github.com/andrada-ianus/TDR_study.git.

## Footnotes

3
https://www.dropbox.com/sh/fx6a04tgpaqva5b/AADpTJi_ZGdbq5N5bwjh1Agja?dl=0

## Data availability

Data will be made available on request.

## References

[R1] Aboitiz F, Scheibel AB, Fisher RS, Zaidel E (1992). Fiber composition of the human corpus callosum. Brain Res.

[R2] Afzali M (2020). Improving neural soma imaging using the power spectrum of the free gradient waveforms. Proc Intl Soc Mag Reson Med.

[R3] Aggarwal M, Smith MD, Calabresi PA (2020). Diffusion-time dependence of diffusional kurtosis in the mouse brain. Magn Reson Med.

[R4] Alexander DC (2010). Orientationally invariant indices of axon diameter and density from diffusion MRI. Neuroimage.

[R5] Alexander DC, Dyrby TB, Nilsson M, Zhang H (2019). Imaging brain microstructure with diffusion MRI: practicality and applications. NMR Biomed.

[R6] Alves R (2022). Correlation Tensor MRI deciphers underlying kurtosis sources in stroke. Neuroimage.

[R7] Anaby D (2019). Single-and double-Diffusion encoding MRI for studying ex vivo apparent axon diameter distribution in spinal cord white matter. NMR Biomed.

[R8] Assaf Y, Mayk A, Cohen Y (2000). Displacement imaging of spinal cord using q-space diffusion-weighted MRI. Magn Reson Med.

[R9] Assaf Y, Blumenfeld-Katzir T, Yovel Y, Basser PJ (2008). AxCaliber: a method for measuring axon diameter distribution from diffusion MRI. Magn Reson Med.

[R10] Baliyan V, Das CJ, Sharma R, Gupta AK (2016). Diffusion weighted imaging: technique and applications. World J Radiol.

[R11] Bar-Shir A, Cohen Y (2008). High b-value q-space diffusion MRS of nerves: structural information and comparison with histological evidence. NMR Biomed.

[R12] Barazany D, Basser PJ, Assaf Y (2009). *In vivo* measurement of axon diameter distribution in the corpus callosum of rat brain. Brain.

[R13] Bezchlibnyk YB (2007). Neuron somal size is decreased in the lateral amygdalar nucleus of subjects with bipolar disorder. J Psychiatry Neurosci.

[R14] Budde MD, Frank JA (2010). Neurite beading is sufficient to decrease the apparent diffusion coefficient after ischemic stroke. Proc Natl Acad Sci U S A.

[R15] Callaghan PT, Jolley KW, Lelievre J (1979). Diffusion of water in the endosperm tissue of wheat grains as studied by pulsed field gradient nuclear magnetic resonance. Biophys J.

[R16] Callaghan R, Alexander DC, Palombo M, Zhang H, ConFiG (2020). Contextual Fibre Growth to generate realistic axonal packing for diffusion MRI simulation. Neuroimage.

[R17] Callaghan R, Alexander DC, Palombo M, Zhang H (2021). Impact of within-voxel heterogeneity in fibre geometry on spherical deconvolution.

[R18] Callaghan PT (1997). A simple matrix formalism for spin echo analysis of restricted diffusion under generalized gradient waveforms. J Magn Reson.

[R19] Cluskey S, Ramsden DB (2001). Mechanisms of neurodegeneration in amyotrophic lateral sclerosis. Mol Pathol.

[R20] Dell’Acqua F (2019). Temporal diffusion ratio (TDR): a diffusion MRI technique to map the fraction and spatial distribution of large axons in the living human brain.

[R21] Devan SP (2022). Selective cell size MRI differentiates brain tumors from radiation necrosis. Cancer Res.

[R22] Does MD, Parsons EC, Gore JC (2003). Oscillating gradient measurements of water diffusion in normal and globally ischemic rat brain. Magn Reson Med.

[R23] Drake-Pérez M, Boto J, Fitsiori A, Lovblad K, Vargas MI (2018). Clinical applications of diffusion weighted imaging in neuroradiology. Insights Imaging.

[R24] Drobnjak I, Siow B, Alexander DC (2010). Optimizing gradient waveforms for microstructure sensitivity in diffusion-weighted MR. J Magn Reson.

[R25] Drobnjak I, Zhang H, Hall MG, Alexander DC (2011). The matrix formalism for generalised gradients with time-varying orientation in diffusion NMR. J Magn Reson.

[R26] Drobnjak I, Zhang H, Ianuş A, Kaden E, Alexander DC (2016). PGSE, OGSE, and sensitivity to axon diameter in diffusion MRI: insight from a simulation study. Magn Reson Med.

[R27] Dubois J (2014). The early development of brain white matter: a review of imaging studies in fetuses, newborns and infants. Neuroscience.

[R28] Dukkipati SS, Garrett TL, Elbasiouny SM (2018). The vulnerability of spinal motoneurons and soma size plasticity in a mouse model of amyotrophic lateral sclerosis. J Physiol.

[R29] Dula AN, Gochberg DF, Valentine HL, Valentine WM, Does MD (2010). Multiexponential T2 magnetization transfer, and quantitative histology in white matter tracts of rat spinal cord. Magn Reson Med.

[R30] Duval T (2015). In vivo mapping of human spinal cord microstructure at 300mT/m. Neuroimage.

[R31] Duval T (2019). Axons morphometry in the human spinal cord. Neuroimage.

[R32] Eichner C (2015). Real diffusion-weighted MRI enabling true signal averaging and increased diffusion contrast. Neuroimage.

[R33] Fan Q (2020). Axon diameter index estimation independent of fiber orientation distribution using high-gradient diffusion MRI. Neuroimage.

[R34] Fick RHJ, Sepasian N, Pizzolato M, Ianus A, Deriche R (2017). Assessing the feasibility of estimating axon diameter using diffusion models and machine learning.

[R35] Grussu F (2017). Neurite dispersion: a new marker of multiple sclerosis spinal cord pathology? Ann. Clin Transl Neurol.

[R36] Grussu F (2019). Relevance of time-dependence for clinically viable diffusion imaging of the spinal cord. Magn Reson Med.

[R37] Hall MG, Alexander DC (2009). Convergence and parameter choice for Monte-Carlo simulations of diffusion MRI. IEEE Trans Med Imaging.

[R38] Harkins KD, Beaulieu C, Xu J, Gore JC, Does MD (2021). A simple estimate of axon size with diffusion MRI. Neuroimage.

[R39] Heads T, Pollock M, Robertson A, Sutherland WH, Allpress S (1991). Sensory nerve pathology in amyotrophic lateral sclerosis. Acta Neuropathol.

[R40] Houston CM (2017). Exploring the significance of morphological diversity for cerebellar granule cell excitability. Sci Rep.

[R41] Huang SY (2020). High-gradient diffusion MRI reveals distinct estimates of axon diameter index within different white matter tracts in the in vivo human brain. Brain Struct Funct.

[R42] Hursh JB (1939). Conduction velocity and diameter of nerve fibers. Am J Physiol Leg Content.

[R43] Ianuş A, Alexander DC, Drobnjak I (2016). Simulation and Synthesis in Medical Imaging.

[R44] Ianuş A (2022a). Soma and neurite density MRI (SANDI) of the in-vivo mouse brain and comparison with the Allen Brain Atlas. Neuroimage.

[R45] Ianus A, Alexander DC, Zhang H, Palombo M (2021). Mapping complex cell morphology in the grey matter with double diffusion encoding MR: a simulation study. Neuroimage.

[R46] Ianus A, Cruz R, Chavarrias C, Palombo M, Shemesh N (2022b). Early microstructural aberrations in a mouse model of Alzheimer’s disease detected by Soma and Neu-rite Density Imaging. Proc Intl Soc Mag Reson Med.

[R47] Jelescu IO, Veraart J, Fieremans E, Novikov DS (2016). Degeneracy in model parameter estimation for multi-compartmental diffusion in neuronal tissue. NMR Biomed.

[R48] Jelescu IO, de Skowronski A, Geffroy F, Palombo M, Novikov DS (2022). Neurite Exchange Imaging (NEXI): a minimal model of diffusion in gray matter with inter-compartment water exchange. Neuroimage.

[R49] Jespersen SN, Olesen JL, Hansen B, Shemesh N (2018). Diffusion time dependence of microstructural parameters in fixed spinal cord. Neuroimage.

[R50] Kakkar LS (2018). Low frequency oscillating gradient spin-echo sequences improve sensitivity to axon diameter stud y i: an experimental n viable nerve tissue. Neuroimage.

[R51] Lackey EP, Heck DH, Sillitoe RV (2018). Recent advances in understanding the mechanisms of cerebellar granule cell development and function and their contribution to behavior. F1000Res.

[R52] Lampinen B, Lätt J, Wasselius J, van Westen D, Nilsson M (2021). Time dependence in diffusion MRI predicts tissue outcome in ischemic stroke patients. Magn Reson Med.

[R53] Lawrence KE (2021). Age and sex effects on advanced white matter microstructure measures in 15,628 older adults: a UK biobank study. Brain Imaging Behav.

[R54] Le Bihan D, Iima M (2015). diffusion magnetic resonance imaging: what water tells us about biological tissues. PLoS Biol.

[R55] Lebel C, Caverhill-Godkewitsch S, Beaulieu C (2010). Age-related regional variations of the corpus callosum identified by diffusion tensor tractography. Neuroimage.

[R56] Lee H-H, Papaioannou A, Kim S-L, Novikov DS, Fieremans E (2020a). A time-dependent diffusion MRI signature of axon caliber variations and beading. Communications Biology.

[R57] Lee H-H, Jespersen SN, Fieremans E, Novikov DS (2020b). The impact of realistic axonal shape on axon diameter estimation using diffusion MRI. Neuroimage.

[R58] Neuman CH (1974). Spin echo of spins diffusing in a bounded medium. J Chem Phys.

[R59] Nilsson M, Lätt J, Ståhlberg F, van Westen D, Hagslätt H (2012). The importance of axonal undulation in diffusion MR measurements: a Monte Carlo simulation study. NMR Biomed.

[R60] Nilsson M, Lasič S, Drobnjak I, Topgaard D, Westin C-F (2017). Resolution limit of cylinder diameter estimation by diffusion MRI: the impact of gradient waveform and orientation dispersion. NMR Biomed.

[R61] Novikov DS, Kiselev VG, Jespersen SN (2018). On modeling. Magn Reson Med.

[R62] Nunes D, Cruz TL, Jespersen SN, Shemesh N (2017). Mapping axonal density and average diameter using non-monotonic time-dependent gradient-echo MRI. J Magn Reson.

[R63] Olesen JL, Østergaard L, Shemesh N, Jespersen SN (2021). Beyond the diffusion standard model in fixed rat spinal cord with combined linear and planar encoding. Neuroimage.

[R64] Olesen JL, Østergaard L, Shemesh N, Jespersen SN (2022a). Diffusion time dependence, power-law scaling, and exchange in gray matter. Neuroimage.

[R65] Olesen JL, Ianus A, Shemesh N, Jespersen SN (2022b). Time dependence at ultra-high diffusion weighting reveals fast compartmental exchange in rat cortex in vivo. Proc Intl Soc Mag Reson Med.

[R66] Ong HH, Wehrli FW (2010). Quantifying axon diameter and intra-cellular volume fraction in excised mouse spinal cord with q-space imaging. Neuroimage.

[R67] Palombo M (2020). SANDI: a compartment-based model for non-invasive apparent soma and neurite imaging by diffusion MRI. Neuroimage.

[R68] Palombo M, Alexander DC, Zhang H (2021). Large-scale analysis of brain cell morphometry informs microstructure modelling of gray matter.

[R69] Panagiotaki E (2014). Noninvasive quantification of solid tumor microstructure using VERDICT MRI. Cancer Res.

[R70] Paquette M, Eichner C, Knösche TR, Anwander A (2021). Axon diameter measurements using diffusion MRI are infeasible. bioRxiv.

[R71] Rajkowska G, Selemon LD, Goldman-Rakic PS (1998). Neuronal and glial somal size in the prefrontal cortex: a postmortem morphometric study of schizophrenia and Huntington disease. Arch Gen Psychiatry.

[R72] Reynaud O (2017). Time-dependent diffusion MRI in cancer: tissue modeling and applications. Front Phys.

[R73] Ritchie JM (1982). On the relation between fibre diameter and conduction velocity in myelinated nerve fibres. Proc R Soc Lond B Biol Sci.

[R74] Roberts TA (2020). Noninvasive diffusion magnetic resonance imaging of brain tumour cell size for the early detection of therapeutic response. Sci Rep.

[R75] Schiavi S (2022). Dissecting brain grey and white matter microstructure: a novel clinical diffusion MRI protocol. bioRxiv.

[R76] Sepehrband F, Alexander DC, Kurniawan ND, Reutens DC, Yang Z (2016). Towards higher sensitivity and stability of axon diameter estimation with diffusion-weighted MRI. NMR Biomed.

[R77] Sexton CE (2014). Accelerated changes in white matter microstructure during aging: a longitudinal diffusion tensor imaging study. J Neurosci.

[R78] Shemesh N, Álvarez GA, Frydman L (2015). size distribution imaging by non-uniform oscillating-gradient spin echo (NOGSE) MRI. PLoS One.

[R79] Shemesh N (2018). Axon diameters and myelin content modulate microscopic fractional anisotropy at short diffusion times in fixed rat spinal cord. Front Phys.

[R80] Siow (2013). Axon radius estimation with oscillating gradient spin echo (OGSE) diffusion MRI. Diffus Fundam.

[R81] Skinner NP, Kurpad SN, Schmit BD, Budde MD (2015). Detection of acute nervous system injury with advanced diffusion-weighted MRI: a simulation and sensitivity analysis. NMR Biomed.

[R82] Stanisz GJ, Szafer A, Wright GA, Henkelman RM (1997). An analytical model of restricted diffusion in bovine optic nerve. Magn Reson Med.

[R83] Taylor DC, Falconer MA, Bruton CJ, Corsellis JA (1971). Focal dysplasia of the cerebral cortex in epilepsy. J Neurol Neurosurg Psychiatry.

[R84] Tian L, Ma L (2017). Microstructural changes of the human brain from early to mid-adulthood. Front Hum Neurosci.

[R85] Tian Q (2022). Comprehensive diffusion MRI dataset for in vivo human brain microstructure mapping using 300 mT/m gradients. Sci Data.

[R86] van Gelderen P, DesPres D, van Zijl PC, Moonen CT (1994). Evaluation of restricted diffusion in cylinders. Phosphocreatine in rabbit leg muscle J Magn Reson B.

[R87] Veraart J (2016). Denoising of diffusion MRI using random matrix theory. Neuroimage.

[R88] Veraart J, Fieremans E, Novikov DS (2019). On the scaling behavior of water diffusion in human brain white matter. Neuroimage.

[R89] Veraart J (2020). Noninvasive quantification of axon radii using diffusion MRI. Elife.

[R90] Watson GS (1965). equatorial distributions on a sphere. Biometrika.

[R91] Wu D, Martin LJ, Northington FJ, Zhang J (2014). Oscillating gradient diffusion MRI reveals unique microstructural information in normal and hypoxia-ischemia injured mouse brains. Magn Reson Med.

[R92] Wu D, Martin LJ, Northington FJ, Zhang J (2019). Oscillating-gradient diffusion magnetic resonance imaging detects acute subcellular structural changes in the mouse forebrain after neonatal hypoxia-ischemia. J Cereb Blood Flow Metab.

[R93] Xu J (2014). Mapping mean axon diameter and axonal volume fraction by MRI using temporal diffusion spectroscopy. Neuroimage.

[R94] Xu J (2016). Fast and simplified mapping of mean axon diameter using temporal diffusion spectroscopy. NMR Biomed.

[R95] Zhang H, Dyrby TB, Alexander DC (2011). Axon diameter mapping in crossing fibers with diffusion MRI. Med Image Comput Comput Assist Interv.

[R96] Zhang H, Schneider T, Wheeler-Kingshott CA, Alexander DC (2012). NODDI: practical*in vivo*neurite orientation dispersion and density imaging of the human brain. Neuroimage.

