# Supplementary material for: Temporal Diffusion Ratio (TDR) for imaging restricted diffusion: Optimisation and pre-clinical demonstration
Source: Neuroimage. Author manuscript; Available in PMC 2023 Oct 26. (PMC7615244; doi:10.1016/j.neuroimage.2023.119930)
Supplement: Appendix 1 [file EMS189813-supplement-Appendix_1.docx]

**Supplementary Material**

***S1. Simulations including a wider range of scanner constraints***

As well as our optimisations in Simulation 1, which use hardware constraints corresponding to a typical pre-clinical scanner, we additionally optimise TDR for G < 2700 mT/m, Δ+δ < 45 ms corresponding to a high-gradient pre-clinical system, G < 300 mT/m, Δ+δ < 80 ms corresponding to the Connectome scanner and G < 80 mT/m, Δ+δ < 80 ms corresponding to a high performance clinical scanner. We keep b = 8 ms/μm^2^. We have also included typical duration for the refocusing pulse between the diffusion gradients, leading to Δ-δ > 2 ms for the preclinical settings and Δ-δ > 7 ms for the clinical settings. Results when optimising TDR for a substrate consisting of large parallel cylinders (as specified in Figure 2) are displayed in Figure S1.

Overall, when optimising TDR for a large cylinder substrate for our four different sets of scanner constraints, we find the following parameter sets:

- G<80mT/m, Δ+δ < 80ms: S_1_: Δ=33.6 ms, δ=26.6 ms; S_2o_: Δ=53.6 ms, δ=26.4 ms
- G<300mT/m, Δ+δ < 80ms: S_1_: Δ=16.6 ms, δ=9.6 ms; S_2o_: Δ=53.6 ms, δ=26.4 ms
- G<600mT/m, Δ+δ < 45ms: S_1_: Δ=8.9 ms, δ=6.9 ms; S_2o_: Δ=31 ms, δ=14.1 ms
- G<2700mT/m, Δ+δ < 45ms: S_1_: Δ=4.2 ms, δ=2.2 ms; S_2o_: Δ=30.9 ms, δ=14.1 ms


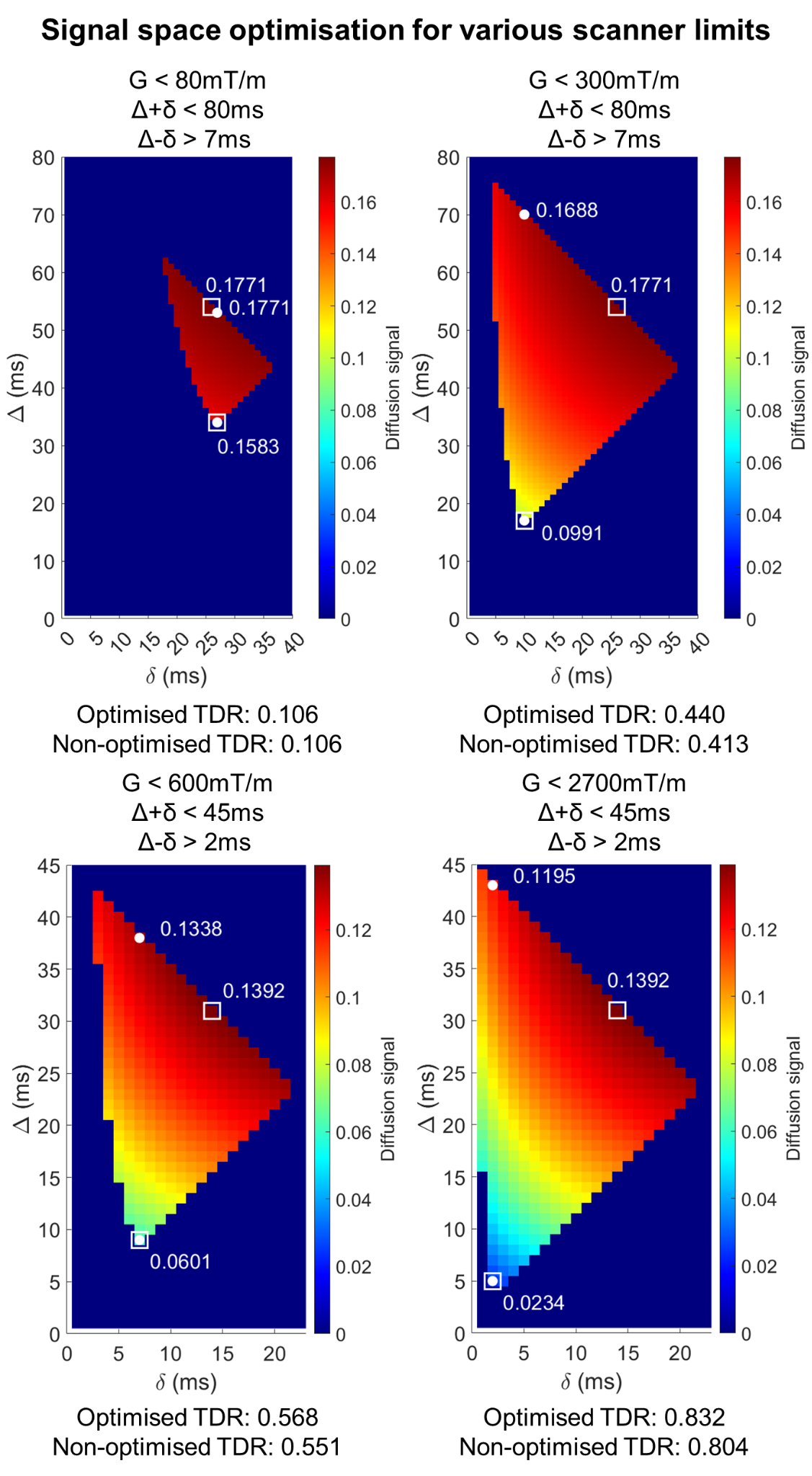


**Figure S1:** Optimisation results maximising TDR for other scanner constraints (b=8 ms/μm^2^). Maps show the diffusion weighted signal for sequences with b=8 ms/μm^2^ and various δ/Δ combinations for the ‘large axon’ substrate illustrated in Figure 2. Signal values are averaged over 60 directions as in the original formulation of TDR. White markers indicate the non-optimised (circle) and optimised (square) sequences, respectively.

The results show that for the lower gradient strengths such as G<80mT/m, signal difference (S_2_ - S_1_) used to calculate TDR is at maximum 0.02, which is extremely small given the noise levels and hence the TDR values calculated for G<80mT/m are neither large enough nor meaningful. Hence, for the TDR method to work, stronger gradients are needed, such as the Connectome Scanner with 300mT/m, which as shown here provides large enough signal difference and TDR values.

***S2. Optimisation including finite slew rates***

Our simulations so far have used rectangular gradient waveform shapes. Whilst this assumption is accurate for the preclinical scanner used in our experiments with a slew rate of 25000mT/ms, the slew rates achievable on clinical scanners are limited due to both hardware limitations and physiological constraints. Therefore, in order to evaluate the effect of this, we perform another optimisation for our Connectome scanner settings with slew rate limited to 200mT/ms, (G < 300 mT/m, Δ+δ < 80 ms, b=8 ms/μm^2^) and compare the results to our infinite-slew optimisation.

We find that the optimal parameters for the gradient waveform assuming slew rate = 200 mT/m are: Δ=17.7 ms, δ=10.7 ms (for the first waveform producing S_1_) and Δ=54 ms, δ=26 ms (for the second waveform producing S_2_). This is extremely close to our original results assuming infinite slew rate: Δ=16.6 ms, δ=9.6 ms (for the first waveform producing S_1_) and Δ=53.6 ms, δ=26.4 ms (for the second waveform producing S_2_) - indicating that the finite slew rate in the optimisation does not significantly change the optimisation results.

***S3. TDR optimisation for sequences with b = 20 ms/μm^2^***

Figures S2 to S4 display the results of our simulations rerun at b = 20 ms/μm^2^, a b-value which will assure minimal signal contribution from extra-axonal space. In Figure S2, we see once again that a gradient waveform with short duration and diffusion time and a gradient waveform with long duration and diffusion time should be contrasted to maximise TDR; we also note that whilst the use of a higher b-value does generally lead to higher TDR values, this is not universally the case, with large cylinders with returning higher TDR values at b = 8 ms/μm^2^ (Figure 3).

Figure S3 shows that broadly speaking the TDR values achievable at b=20 ms/μm^2^are similar to those achievable at b=8 ms/μm^2^ (compare Figure 4).

Figure S4 shows that the decrease in accuracy associated with calculating the TDR of anisotropic structures using a full set of 60 gradient directions is more pronounced at b=20 ms/μm^2^ than at b=8 ms/μm^2^. This highlights the need for calculating TDR using only a small subset of the acquired directions.


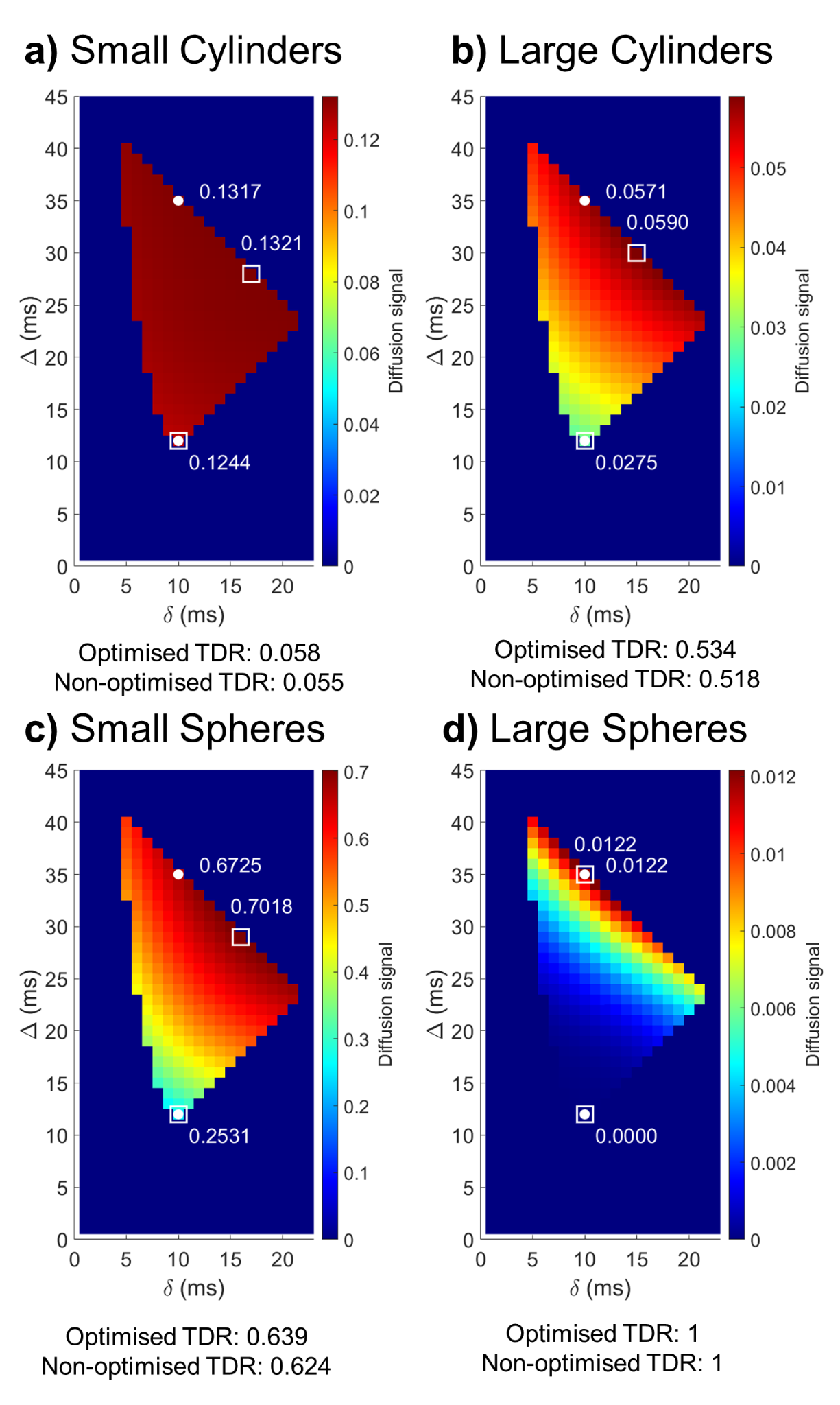


**Figure S2:** Optimisation results maximising TDR, performed at b = 20 ms/μm^2^ with G < 600 mT/m. (a-d) Maps showing the diffusion weighted signal averaged over the 60 uniformly distributed directions for sequences with b=20 ms/μm^2^and various δ/Δ combinations for the substrates illustrated in Figure 2. White markers indicate the non-optimised (circle) and optimised (square) sequences, respectively. The optimised sequences provide larger signal differences between S_1_ and S_2o_ compared to between S_1_ and S_2n_, and higher TDR values.


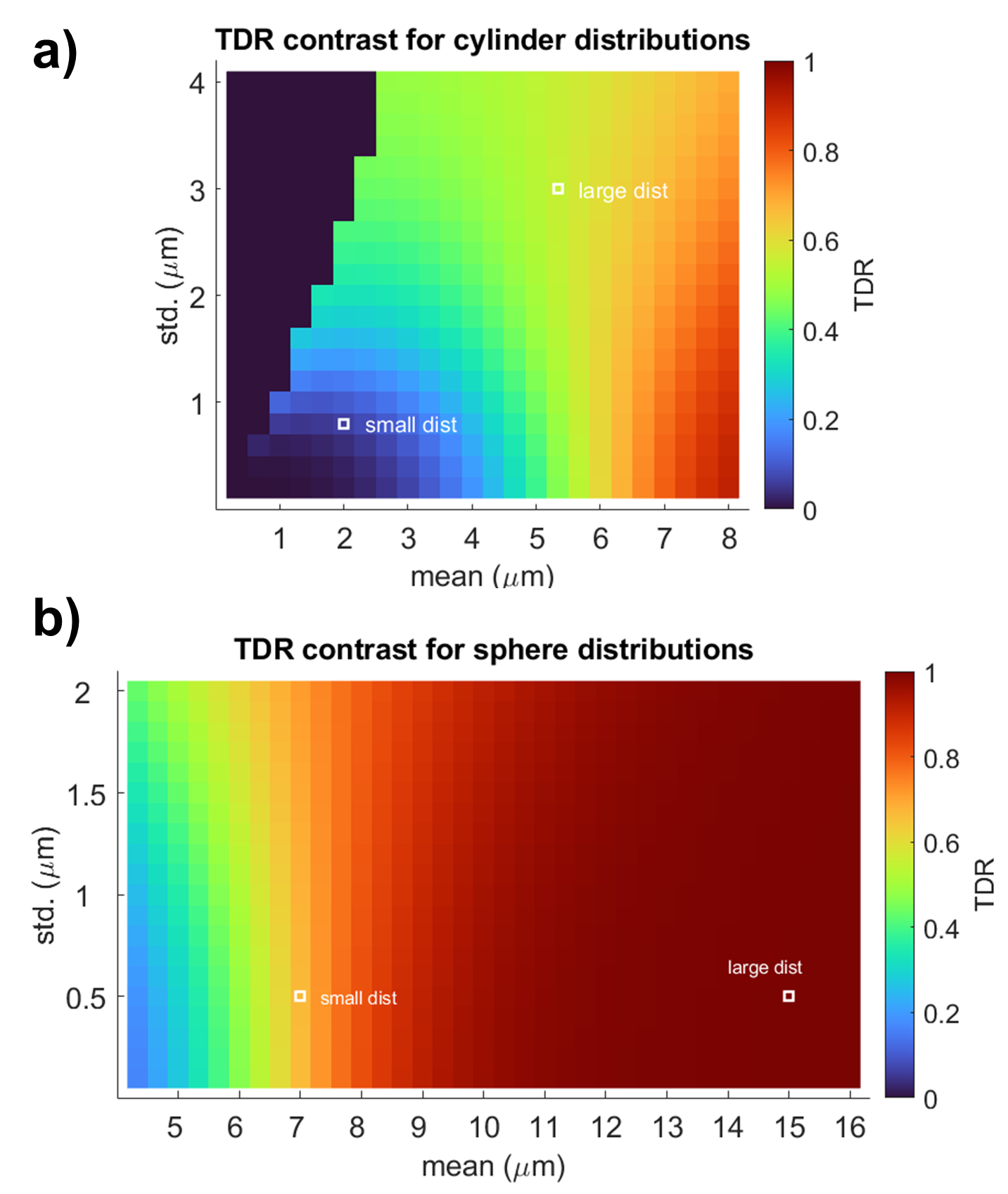


**Figure S3:** Noise-free TDR values calculated for sequences with optimised parameters for b = 20 ms/μm^2^ and G_max_=600 mT/m: S_1_: Δ = 11.61 ms, δ = 9.61 ms; S_2o_: Δ = 29.94 ms, δ = 15.06 ms. The signal is simulated across a wide range of (a) cylinder and (b) spherical diameter distributions. For cylinders, we simulate Gamma distributions, and for spheres we simulate Gaussian distributions, which reflect the size distributions usually measured in the tissue. The typical large and small size distributions presented in Figure 2 are shown using white markers. For cylinders, the gamma distributions are truncated at 20 μm to match realistic values from the tissue; thus, combinations of parameters where this truncation changes the mean by more than 10% were not considered.


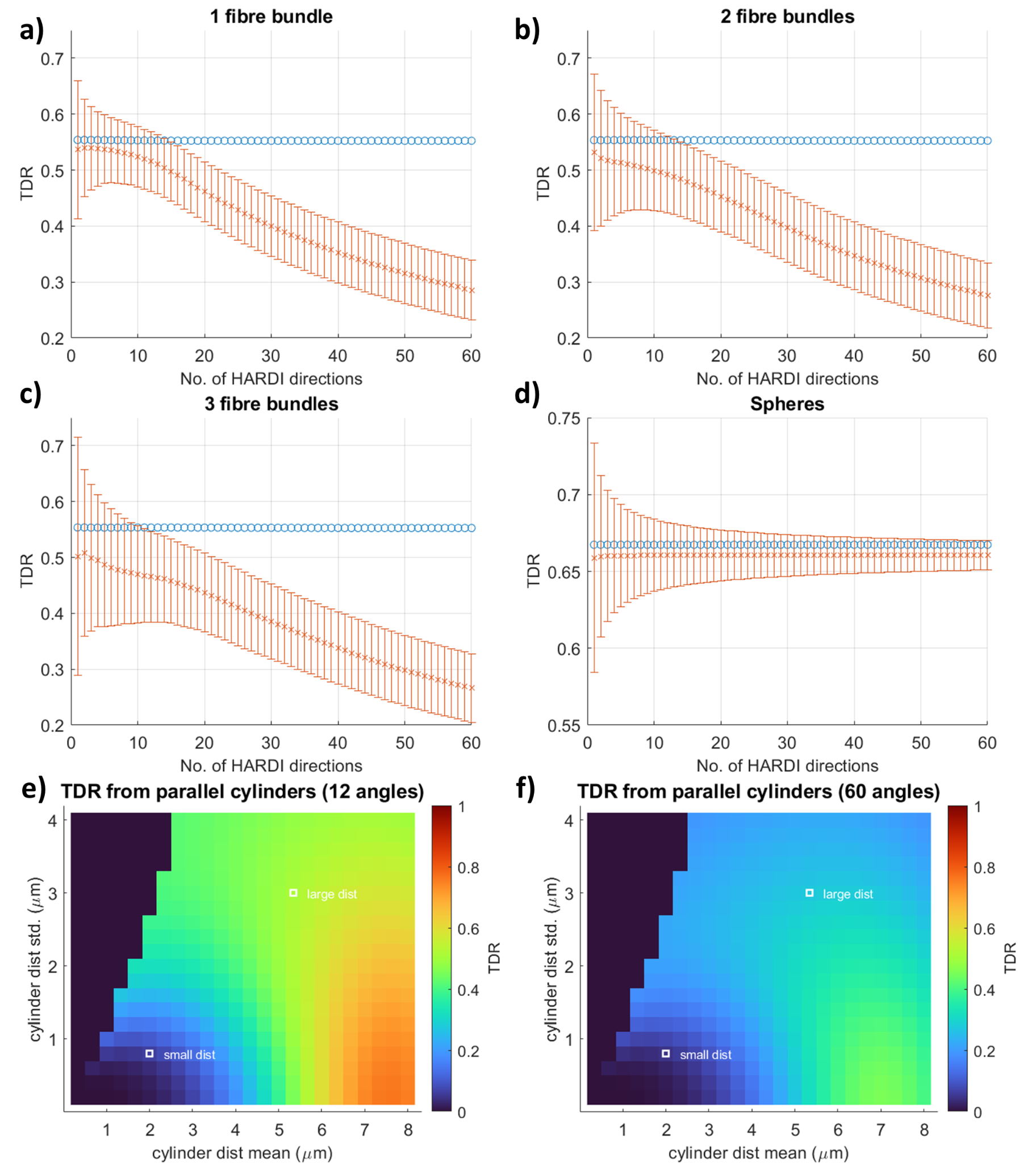


**Figure S4** TDR values at b = 20 ms/μm^2^  for noise free (blue circles, SNR = inf) and noisy (orange crosses, Rician noise with SNR = 20) signals as a function of the number of gradient directions included in the analysis in different substrates: a-c) bundles of cylinders with one, two or three fibre orientations crossing at 90^o^. All bundles have a Gamma distribution of sizes with mean = 5.33 μm and std =3.00 μm; d) a Gaussian distribution of spheres with mean = 7 μm and std = 0.5 μm. The orange error bars show 1 standard deviation of the estimated TDR over 100,000 noise instances. As illustrated in a-c), for the different fibre configurations, TDR values estimated from noisy data are below the expected noise-free values. All simulations are performed using the optimised sequence parameters for large cylinders.

***S4. Exploration of TDR dependence on the number of gradient directions***

As discussed in the results of Simulation 3, calculating TDR using a subset of the acquired gradient directions that provide the highest signals, can increase the accuracy of the TDR estimate in the presence of Rician noise. For the same substrates presented in Figure 6, here we explore this effect for other acquisition scenarios, with different numbers of gradient directions (30 instead of 60) and SNR levels (50 instead of 20).

We find that in all scenarios, calculating TDR using subsets consisting of fewer gradient directions improves the accuracy of TDR (Figure S5).

We also consider the coefficient of variation of the mean TDR in the presence of noise, calculating the coefficient of variation across the three different fibre scenarios. As the three fibre scenarios all include fibres bundles with identical diameter distributions, ideally TDR should return the same value for all three scenarios, and the coefficient of variation should be zero.

We find that when SNR = 50, the coefficient of variation is minimised by using ~33% of the available gradient directions (21/60 and 10/30 directions respectively). Alongside our results for SNR = 50 in Figure S5, this suggests that calculating TDR using a subset of the available directions is optimal.

When SNR = 20 and 60 gradient directions are acquired in total, using 27/60 or 45% is a clear local minimum in the coefficient of variation plot; as the true minimum is found at 2/60 directions, where the variation in calculated TDR due to noise is unacceptably large (Figure 6 a-d) this also suggests using roughly half the gradient directions available is a suitable choice for calculating TDR in the presence of noise.


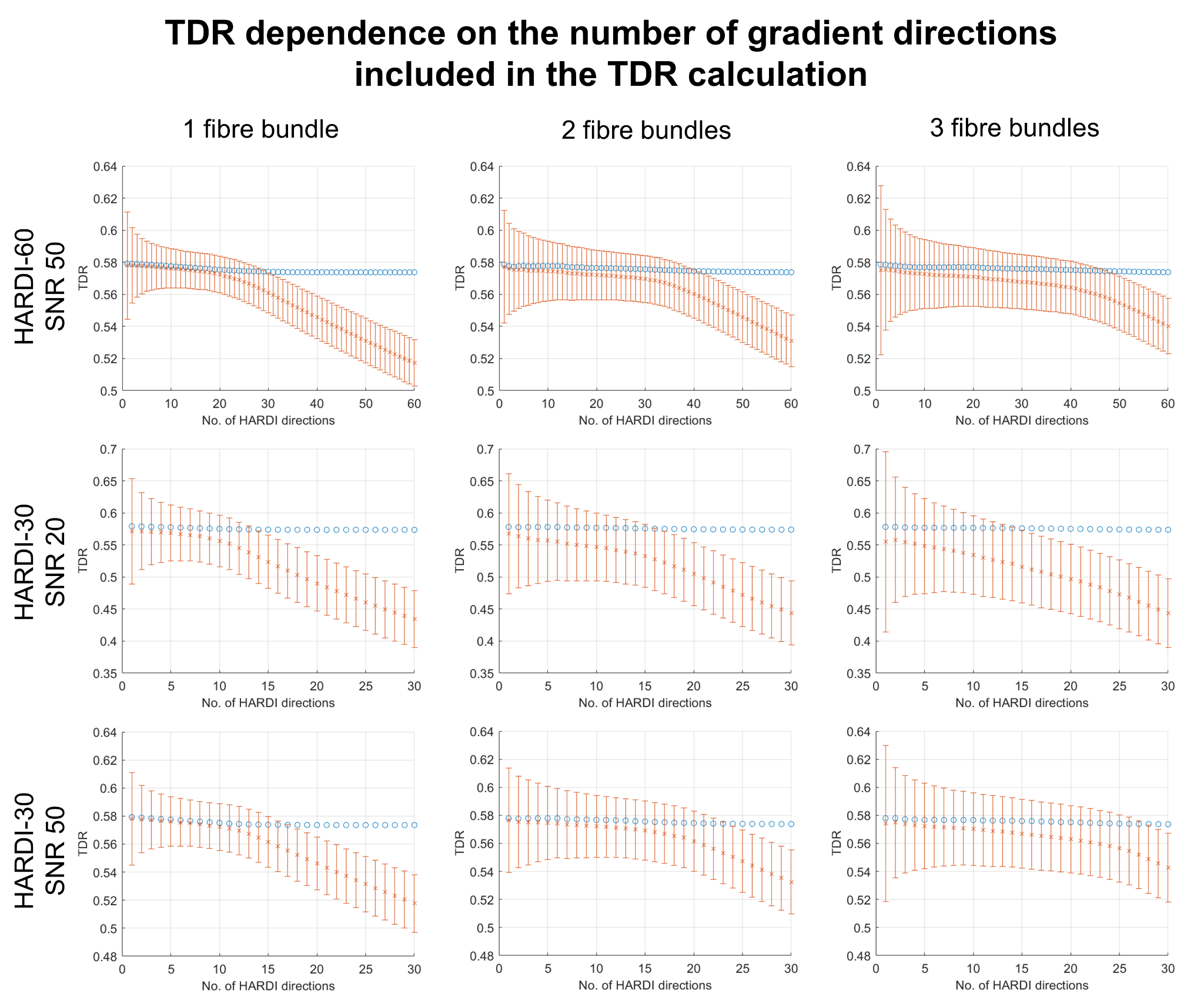


Figure S5: TDR values for noise free (blue circles, SNR = inf) and noisy (orange crosses, Rician noise with SNR as shown) signals as a function of the number of gradient directions included in the analysis, in different substrates consisting of bundles of cylinders with one, two or three fibre orientations crossing at 90^o^ (b=8 ms/μm^2^). All bundles have a Gamma distribution of sizes with mean = 5.33 μm and std =3.00 μm. The orange error bars show 1 standard deviation of the estimated TDR over 100,000 noise instances. All data are acquired using the optimised sequence parameters for large cylinders.


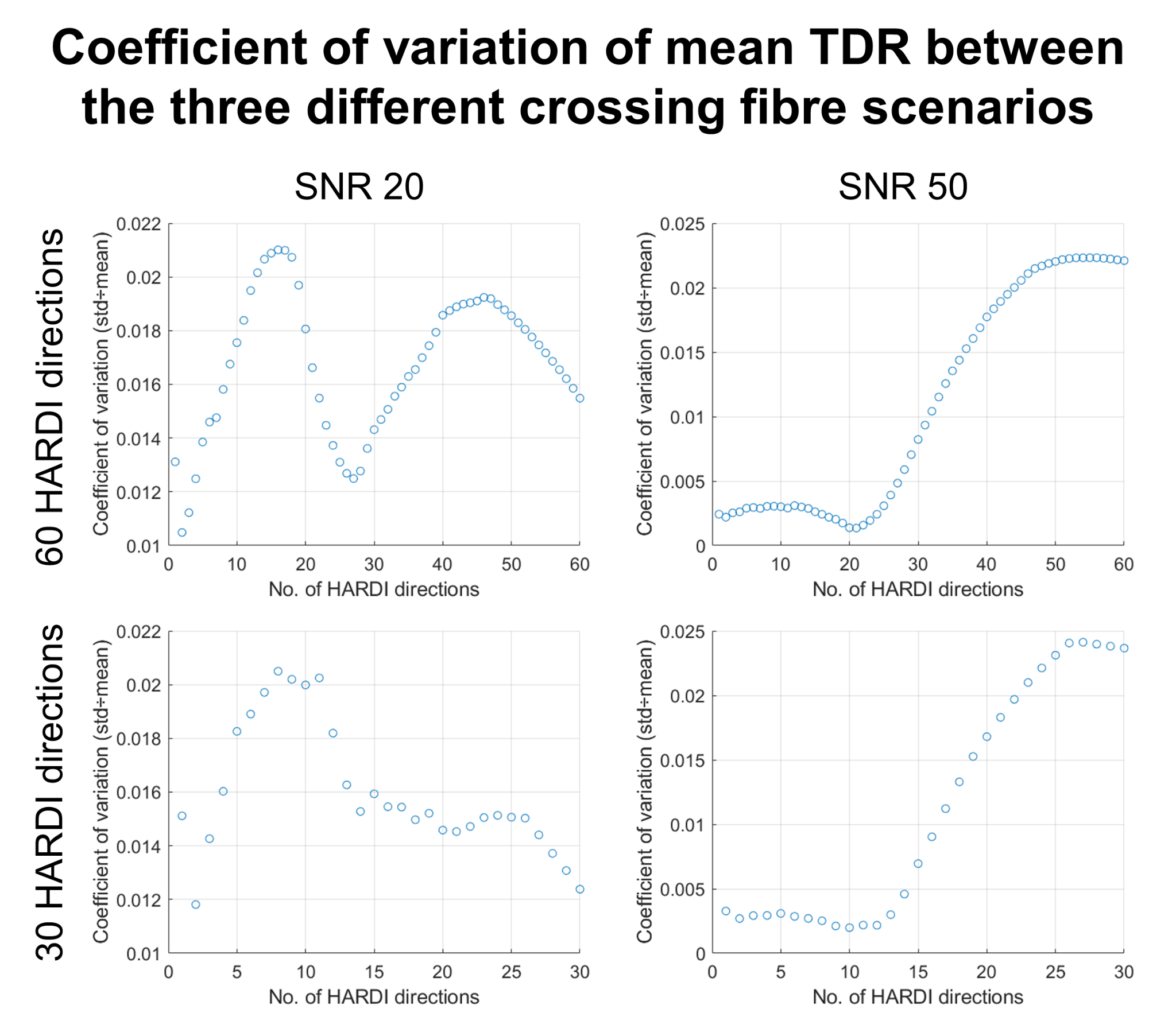


Figure S6: Coefficient of variation of mean TDR between the three scenarios with 1,2 and 3 fibre directions. In simulations with multiple fibre directions the directions chosen are perpendicular. Simulations are run four times, at SNR = 20 and SNR 50 for HARDI shells consisting of 60 and 30 gradient directions.

***S5. Simulations including more realistic substrates and extra-axonal space***

As a proof of concept, we also performed Monte Carlo simulations in substrates generated using the ConFiG framework[^95^](https://paperpile.com/c/SLuBqs/mVOD) that account for dispersion, undulations and diameter variations along the axons. The three substrates considered have different fibre dispersion profiles following a Watson distribution with k = {2, 6, 100}, and a Gamma distribution of the diameters with mean of 2 ± 0.2 μm. A detailed description of the substrates can be found in the recent study by Callaghan et al[^96^](https://paperpile.com/c/SLuBqs/bdS0).

To further improve the realism of the simulations, all metrics are computed as the mean over 100 instances of Rician noise with SNR = 50 added to the simulated signal values.

|  | | High dispersion  diam = 2 ±0.2 μm, f_intra_ = 43%  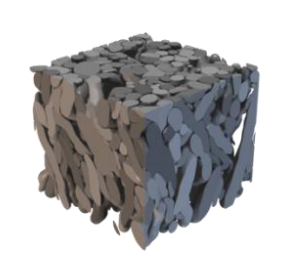 | | Medium dispersion  diam = 2 ±0.2 μm, f_intra_ = 48%  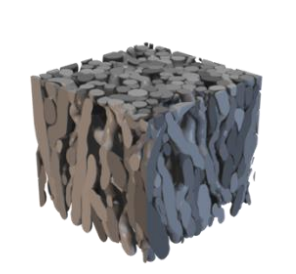 | | Low dispersion  diam = 2 ±0.2 μm, f_intra_ = 58%  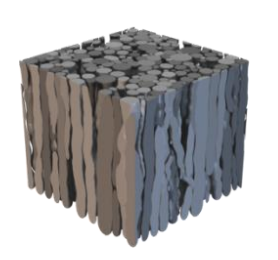 | |
| --- | --- | --- | --- | --- | --- | --- | --- |
|  |  | CONFIG  κ = 2 | Parallel cylinders | CONFIG  κ = 6 | Parallel cylinders | CONFIG  κ = 100 | Parallel cylinders |
| b = 8 ms/μm^2^  G =600 mT/m | Intra | 0.1010 | 0.0099 | 0.0985 | 0.0102 | 0.0698 | 0.0103 |
|  | Uniform | 0.1043 | 0.0012 | 0.1055 | 0.0022 | 0.0738 | 0.0030 |
| b = 8 ms/μm^2^  G =2500 mT/m | Intra | 0.1962 | 0.0627 | 0.1900 | 0.0616 | 0.1437 | 0.0620 |
|  | Uniform | 0.2012 | 0.0310 | 0.1991 | 0.0330 | 0.1550 | 0.0420 |
| b = 20 ms/μm^2^  G =600 mT/m | Intra | 0.1002 | 0.0143 | 0.1017 | 0.0123 | 0.0874 | 0.0138 |
|  | Uniform | 0.1012 | 0.0087 | 0.1026 | 0.0107 | 0.0880 | 0.0121 |
| b = 20 ms/μm^2^  G =2500 mT/m | Intra | 0.2430 | 0.0997 | 0.2409 | 0.0998 | 0.2114 | 0.0936 |
|  | Uniform | 0.2450 | 0.0769 | 0.2428 | 0.0814 | 0.2154 | 0.0845 |

**Table S1** Optimised TDR values (optimised waveforms, 12 gradient directions) for realistic fibre geometries generated using ConFiG and substrates of parallel cylinders with the same intracellular fraction and Gamma distribution of axon diameters. The TDR values are presented for the two b-values and maximum gradient strengths employed in the experimental data. The values are computed based on Monte Carlo simulations with spins distributed either uniformly or only in the intra-axonal compartment.

The TDR values for the preliminary simulations including complex fibre geometries with undulation and variable axon diameter are provided in Table S1. The TDR values for the three substrates generated using the ConFig framework for three different orientation dispersions, specifically Watson distributions with k = 2, 6 and 10, are compared with TDR values from substrates with parallel straight cylinders with the same mean diameter and intra-axonal fraction. For the given mean cylinder diameter (2 ± 0.2 μm), the results show that TDR values are several times higher for the complex fibre geometries compared to straight cylinders, with values around 0.1 for G = 600 mT/m and 0.2 for G = 2500 mT/m.

***S6. TDR in rat spinal cord at b = 20 ms/μm^2^***

Figure S7 presents the TDR analysis in the ex-vivo rat spinal cord for sequences with b = 20 ms/μm^2^.


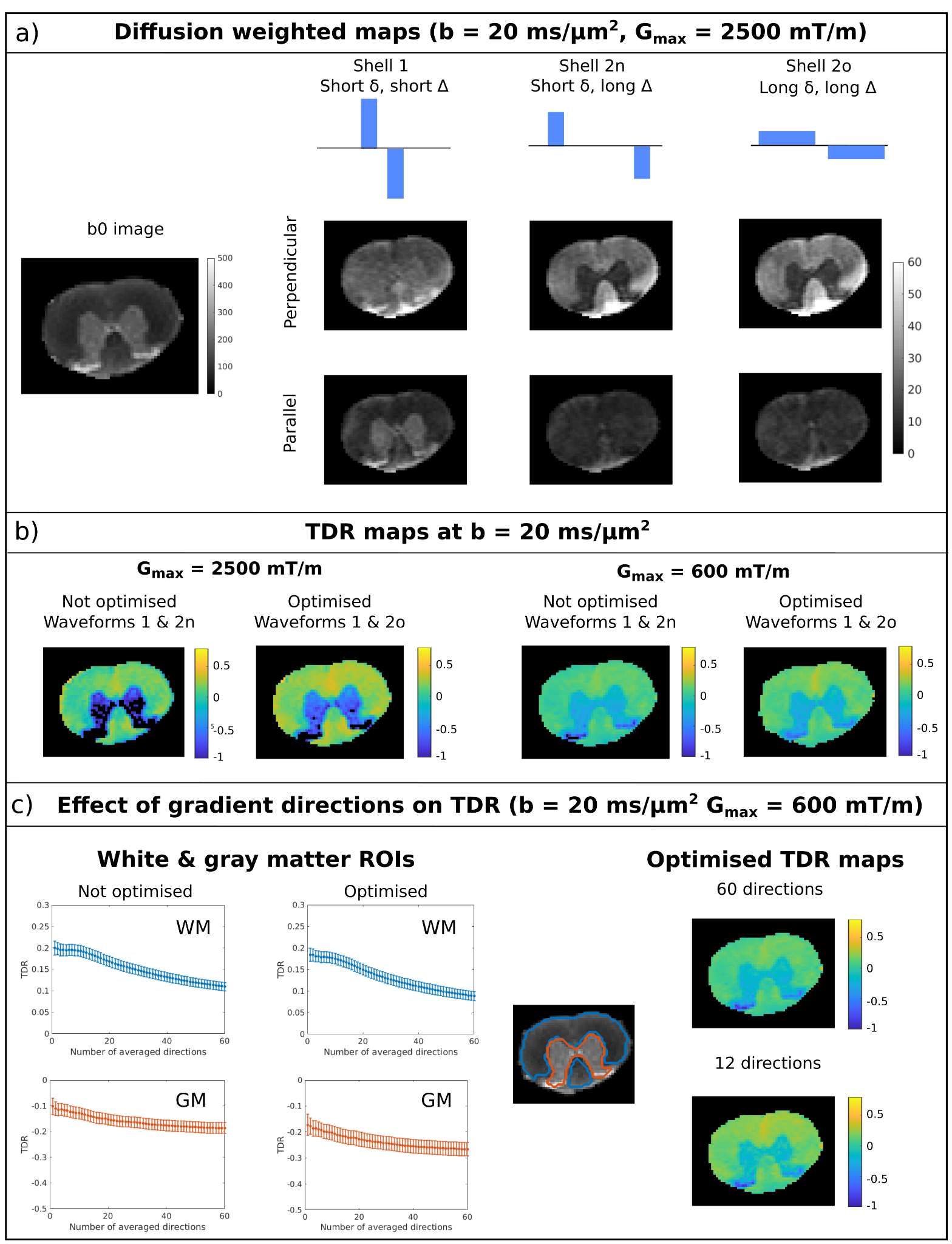


**Figure S7.** Optimisation of TDR acquisition at b = 20 ms/μm^2^. including waveforms (a-b) and number of gradient directions (b). a) left: T2 weighted image of the rat spinal cord without diffusion gradients. White matter and gray matter regions are delineated with blue and orange contours, respectively. Right: schematic depiction of the gradient waveforms for the three shells (top) and the corresponding diffusion weighted maps for a perpendicular (middle) and a parallel (bottom) gradient direction. b) TDR maps for maximum gradient strength of 600 and 2500 mT/m for optimised and non-optimised gradient waveforms. c) left: TDR values as a function of how many gradient directions were included in the signal average for white matter (blue) and gray matter (orange) ROIs. right: TDR maps for measurement subsets with 12/60 and 60/60 directions.
